# Development of robust dual functioning PPy-based photothermal membranes for simultaneous freshwater and salt harvesting

**DOI:** 10.1038/s41598-026-35812-y

**Published:** 2026-02-10

**Authors:** Mahmoud Taha Mahmoud, Hamdy Maamoun Abdel-Ghafar, Ahmed Abdou El-Sherif, Mohamed Saada El-Deab

**Affiliations:** 1 Central Metallurgical Research and Development Institute (CMRDI), PO Box 87, Helwan, Cairo, Egypt; 2 Department of Chemistry, Faculty of Science, Cairo University, Cairo, 12613 Egypt

**Keywords:** Brine, Salt harvesting, Sustainable desalination, Zero liquid discharge, CVDP, Chemistry, Energy science and technology, Engineering, Environmental sciences, Materials science

## Abstract

Photothermal membranes driven by solar energy represent a promising approach for sustainable and cost-effective desalination and wastewater treatment. Solar energy’s renewability and low environmental impact drive its adoption in desalination technologies. In this work, chemical vapor deposition polymerization (CVDP) is used to deposit high-performance polypyrrole (PPy) coatings on two different fabric types: woven and nonwoven, enabling broad-spectrum solar light absorption and efficient thermal conversion for enhanced water evaporation. This study investigated the efficacy of different oxidizing agents in initiating the CVDP process and in depositing PPy layer on the substrate surface, for use as a photothermal membrane to harvest freshwater and salt from different saline wastewater samples. The goal is to achieve efficient polymerization, targeting pyrrole usage as low as 15 μL for perfect PPy deposition. Among the investigated oxidizing agents, copper chloride and ammonium persulfate yielded the most effective performance in producing PPy-coated photothermal membranes. These membranes demonstrated superior light absorption and achieved surface temperatures of 63 °C and 60 °C under simulated 1 sun illumination (1 kW m^− 2^), showing facilitated enhanced water evaporation rates of 0.95 and 0.93 kg m^− 2^ h^− 1^, respectively. Meanwhile, a a water evaporation rate of 2.91 kg m^− 2^ h^− 1^ under 3 sun illumination was obtained. Furthermore, the developed robust photothermal membranes tested against different saline solutions, including NaCl, CuSO_4_.5H_2_O, and FeCl_3_ for simultaneous water evaporation and salt harvesting under solar simulator. A developed A4-size photothermal membrane from non-woven fabric was tested using actual brine water sample under natural sunlight for one week. The used photothermal membranes showed the recyclability and durability during the tests.

**Supplementary Information:**

The online version contains supplementary material available at 10.1038/s41598-026-35812-y.

## Introduction

Population growth and increasing pollution are disrupting the availability of freshwater resources, and hindering the path towards sustainable development^1,2^. However, conventional desalination approaches present environmental drawbacks or require significant energy inputs, hindering their sustainability^3^. Therefore, green renewable energy sources are emerging as a promising avenue for powering sustainable desalination technologies. Solar-driven interfacial evaporation represents a promising approach for sustainable desalination and wastewater treatment. This technology offers several advantages, including minimal reliance on conventional energy sources and a reduced environmental footprint^4^.

In recent years, significant research efforts have been devoted to developing high-performance photothermal materials for light-absorbing layers in desalination membranes. These efforts have explored a diverse range of materials, including noble metal nanoparticles^5,6^, carbon-based materials^8^, natural materials, polymers^]^, and aerogels^]^. The development of high-efficiency photothermal materials for solar-driven evaporation prioritizes two key objectives: (i) maximizing the light absorption efficiency and (ii) minimizing the thermal energy loss^]^. Engineered micro-structures with tailored surface roughness demonstrably enhance light absorption by minimizing light scattering. This approach leads to superior solar energy harvesting efficiency^15,16^. Titanium nitride (TiN)-modified carbon fiber fabric demonstrated a remarkable water evaporation rate of 1.82 kg m^− 2^ h^− 1^ under simulated 1 sun illumination, achieving an exceptional light absorption efficiency of up to 93.4%^17^. On the other hand, evaporators fabricated using carbonized natural materials achieved a noteworthy water evaporation rate of 1.32 kg m^− 2^ h^− 1^ under simulated 1 sun illumination, demonstrating exceptional light absorption efficiency of up to 97.4%^18^. Beyond the established understanding of polypyrrole’s (PPy) intrinsic properties, a compelling research trajectory is emerging that focuses on its deployment in freshwater generation technologies. Initial exploratory work has provided critical validation for this promising direction. For example, polymerization of PPy inside oil bodies produces fresh water with evaporation rate (2.11 kg m^− 2^ h^− 1^) and evaporation efficiency (92.02%) can be obtained in OB-PPy containing rainwater, similarly^19^. Photothermal cellulose hydrogel microspheres and solar interfacial evaporation to harvest freshwater. The evaporation rate of 2.70 kg m⁻^2^ h⁻^1^ under 1 sun efficiency was obtained^20^. The presented approach not only addresses a key knowledge gap but also paves the way for the scalable adoption of polypyrrole-coated membranes, enhancing their efficacy in sustainable desalination and water purification processes. The ongoing pursuit in photothermal technology centers on identifying materials that possess a unique combination of properties: exceptional light absorption efficiency (photothermal properties), economic viability (lowest cost), superior durability on membrane surfaces (highest stability), and straightforward fabrication methods (ease of preparation).

PPy belongs to the class of conductive polymers, has emerged as a promising material for photothermal evaporation applications^21^. Controllable micro-structures can be engineered onto dark PPy layers deposited via electro-polymerization on various conductive substrates, such as stainless-steel mesh^22^. A major limitation of this method lies in the inherent corrosivity of the target environment, due to the presence of acids, alkalis, or dissolved salts in the water. Fortunately, alternative approaches exist for pyrrole polymerization beyond both chemical and electrochemical processes, offering a broader selection for optimizing PPy characteristics for specific applications^23^. Chemical vapor deposition polymerization (CVDP) offers distinct advantages over other techniques for the fabrication of high-quality PPy thin films. This method enables the creation of conformal films with precise control over their properties, including exceptional uniformity and purity^24^. Building upon the findings of Bui^25^, pyrrole molecules can effectively polymerize within the micro-pores of a metal–organic framework (MOF) via a vapor phase reaction process. In contrast to conventional liquid-phase chemical polymerization, CVDP presents a demonstrably greener synthetic pathway. This claim is substantiated by the process’s intrinsic characteristics—namely its solvent-free and vapor-phase nature—which directly operationalizes the principles of green chemistry by design, eliminating hazardous waste at the source. The vapor-phase mechanism constitutes a fundamental departure from conventional liquid-phase processes, enabling intrinsic waste minimization and obviating the need for hazardous solvents altogether^26^. Utilizing commercial and washable fabric types with a scalable CVDP approach for simultaneous freshwater generation and salts harvesting can solve a big challenge in seawater and brine desalination technologies.

This study investigates the use of various oxidizing agents (NH_4_)_2_S_2_O_8_, FeCl_3_, CuCl_2_, KMnO_4_, and Na_2_Cr_2_O_7_) to initiate the polymerization of pyrrole monomers, forming a PPy light-absorbing layer on the top substrate surfaces. The performance of the resulting photothermal membranes was assessed in terms of evaporation rate, surface temperature, and salt recovery efficiency. Addressing a critical gap in current systems, which often focus solely on freshwater production, this work introduces an innovative dual-output solar steam generation system that enables both high-efficiency water desalination and sustainable salt harvesting—advancing the goals of Zero Liquid Discharge (ZLD). Its versatility was demonstrated through tests with a range of salt solutions, including 3.5% and 7.0% NaCl, FeCl_3_, and CuSO_4_·5H_2_O solutions. The proposed approach offers promising potential for developing next-generation membranes for integrated clean water and resource recovery applications.

## Experimental

### Materials and chemicals

Pyrrole with laboratory reagent grade (assay 97%) was purchased from ADVENT CHEMBIO PVT LTD, India. Ammonium persulfate (APS) (NH_4_)_2_S_2_O_8_) (assay 98%), was purchased from ADVENT CHEMBIO PVT LTD, India. Ferric chloride (FeCl_3_) (assay 99.9%) (extra pure) was purchased from TEKKIM, Tekkim kimya sanayi ve Ti Care Limited Sirketi, Turkey. Copper chloride (CuCl_2_) (assay 99%) was purchased from Scientific for Laboratory Chemical, Egypt. Potassium permanganate (KMnO_4_), (assay 99%) was purchased from dop (DOP ORCANIK KIMYA SAN. VE ITC. LTD. STI, Turkey. Sodium dichromate (Na_2_Cr_2_O_7_), (assay 98%) was purchased from Cambrian Chemicals, Canada. Hydrochloric acid (HCl) (assay 30–34%) was purchased from Adwic, El Nasr Pharmaceutical Company, Egypt. Sodium chloride (NaCl) (assay 98.5%) was purchased from Hana Salt (Commercial Salt) Egypt. Ethanol (assay 99.8%) was purchased from Honeywell, Riedel–de Haen, Germany. Commercial-grade of CuSO_4_.5H_2_O was purchased from the local market in Egypt. All chemicals were used as-received without additional purification. Bi-distilled water was used in all the experiments to prepare the various brines. Two chemically resistant commercial filter cloths were used, polyester sheet (non-woven fabric), and woven fabric.

### Methods of optimizing different oxidizing agents with CVDP of pyrrole

**Fig. 1:**
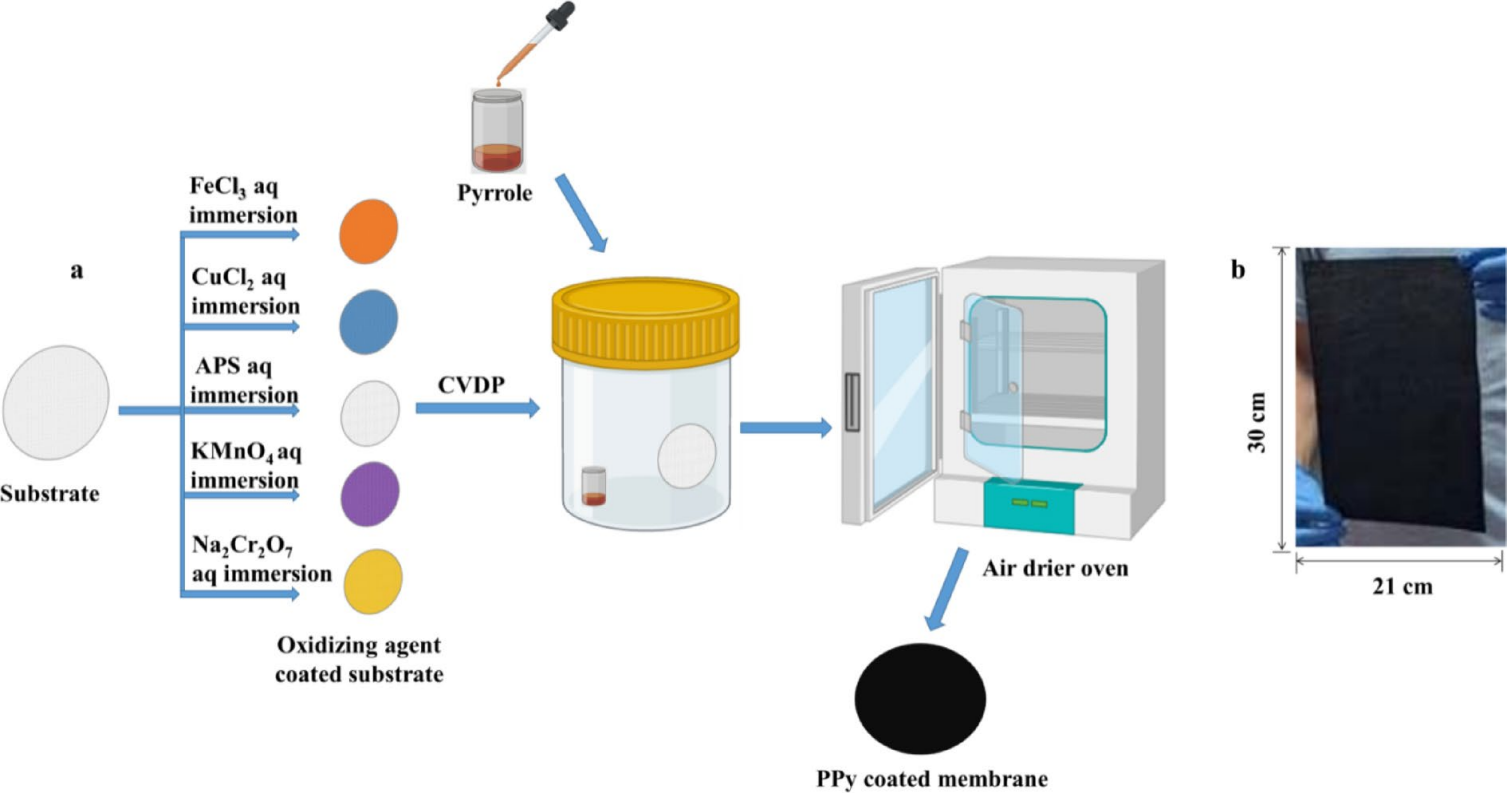
(**a**) Schematic illustration of preparing and developing photothermal membranes using different oxidizing agents. (**b**) Real photographic image of the prepared A4-size photo-thermal membrane from non-woven fabric.

The chemical vapor deposition polymerization (CVDP) of pyrrole was optimized at different pyrrole concentrations (0–25 µL) using two types of porous substrates (woven and non-woven fabric) which were made of polyester. The dense, oriented structure of polyester woven fabric provides high chemical resistance, effectively resisting hydrolytic degradation. This enhanced durability improved the membrane’s efficacy for salt extraction, even under conditions of severe chemical corrosivity. It proved to be highly stable, with no observed degradation, and consistently reproducible performance, confirming its suitability for such demanding applications, preserving the core filament strength. The voluminous pore structure of the non-woven fabric with lower thickness creates a much more open pathway for water vapor molecules to diffuse through which produces the highest evaporation rate, with different oxidizing agents (NH_4_)_2_S_2_O_8_, FeCl_3_, CuCl_2_, KMnO_4,_ and Na_2_Cr_2_O_7_), at different concentrations. Firstly, the substrates were cut to a circular shape with a diameter of 40 mm. Then, the porous substrate was immersed in the oxidizing agent solution (0.1 and 0.5 M) for a certain time. Soaking time was based on the type of oxidizing agent, e.g., the prepared substrates using FeCl_3_ were immersed in the solution for 6 h and subsequently dried at 70°C for 30 min. In contrast, for ammonium persulfate (APS), copper chloride (CuCl_2_), potassium permanganate (KMnO_4_), and sodium dichromate (Na_2_Cr_2_O_7_), the immersion time is reduced to 30 min, followed by drying at room temperature for 15 min. The application parameters, specifically immersion time and drying temperature, were optimized according to the unique behavior of each oxidizing agent. Ferric chloride, which exhibits poor adsorption kinetics, required a prolonged immersion time to achieve adequate surface loading. This was followed by a moderate thermal treatment at 70°C to immobilize the agent. Conversely, alternative oxidizers with high adsorption affinity were processed without thermal drying to prevent their thermal decomposition, a limitation not encountered with the more stable ferric chloride. The oxidizing agent–containing substrate was sealed in a plastic container (PC). Different PC types, adapted for microwave and dryer use, were employed. Pyrrole was added in varying volumes (0, 5, 10, 15, 20, and 25 μL) to enable in situ polymerization at 80°C for 1 h. This exposure triggers in-situ polymerization at an elevated temperature of 80°C. Visual inspection reveals that the surface of the substrate exposed to the pyrrole vapor exhibits a darker coloration compared to the opposing surface that was in contact with the vessel wall. The polymerization process was spatially controlled by exposing a single side of the membrane to pyrrole vapor, thereby localizing polypyrrole deposition. This fabrication method yields a functionally graded membrane, characterized by a hydrophobic polypyrrole layer on one side and an inherent hydrophilic substrate on the other. The deliberate retention of the hydrophilic surface serves to accelerate pumping of water to the membrane. This means the formation of a PPy dark layer on the top surface of the substrate. This top surface side is the main exposing and irradiate by light source in the photothermal membrane while the bottom side is in contact with water source. As a final step, the substrates are thoroughly washed. The post-polymerization washing protocol is dependent on the oxidizing agent employed. For substrates treated with iron (III) chloride (FeCl_3_), a sequential rinse with 0.5 M HCl, ethanol, and deionized (DI) water is required, repeated multiple times for thorough cleaning. In contrast, substrates processed with ammonium persulfate (APS), copper (II) chloride (CuCl_2_), KMnO_4_, and Na_2_Cr_2_O_7_ require only multiple washes with copious amounts of DI water to remove any residues of oxidizing agents or pyrrole. After washing, samples were dried at 60°C for 12 h. Figure 1. illustrates the developed methodology of photothermal membrane fabrication using different oxidizing agents.

The selection of PPy as the photothermal layer was deliberate, based on its unique combination of properties essential for efficient solar-driven evaporation. While considerations regarding monomer cost and synthesis complexity are acknowledged, PPy offers several compelling advantages. Primarily, PPy exhibits exceptional broadband solar absorption and a high photothermal conversion efficiency, often exceeding that of other polymers and even some metallic nanoparticles. Furthermore, the in-situ polymerization of PPy allows for the facile, uniform, and conformal coating of low-cost, flexible substrates such as the polyester fabric used in this study. This process is more straightforward and scalable than the fabrication of membranes incorporated with fragile inorganic nano-materials. Finally, the inherent hydrophilicity and chemical stability of PPy synergize with the polyester substrate to promote rapid water transport and durable operation in harsh environments, making it a superior choice for practical applications^27^.

Although PPy has a relatively high cost, its synthesis was conducted under atmospheric conditions by low-cost methodology of CVDP using inexpensive monomers (pyrrole) and oxidants, making it suitable for laboratory-scale and potential scalable production.

#### Characterization

The surface morphology of the prepared photothermal membranes was characterized using scanning electron microscopy (SEM, Thermo Scientific Prisma E, USA), (FESEM). Fourier transform infrared (FTIR) spectra were acquired using a Vertex 70 RAM II FTIR spectrometer. X-ray diffraction (XRD) spectra were acquired using (XRD, Cu Kα radiation, K = 1.54178 Å, D/max 2550 V, Rigaku, Japan). Static contact angle measurements were performed using a contact angle analyzer (KRÜSS GmbH, DSA25B). UV–vis-near infrared (UV–vis-NIR) absorption spectra were recorded in the 200–2400 nm wavelength range using a PerkinElmer Lambda 1050 spectrophotometer. Both transmittance and diffuse reflectance measurements were obtained using an integrating sphere unit coupled with an automated reflectance measurement system. The measurements were subjected to a multi-step correction process. This included baseline correction to remove instrumental drift, and potentially, background subtraction using a dark spectrum (depending on the specific measurement type). Thermal images were acquired using a FLIR infrared camera. The surface roughness of the prepared photothermal membranes was investigated using Flexaxiom Nanosurf AFM, non- contact mode, NCLR cantilever at room temperature.

### Evaporation performance measurement

**Fig. 2:**
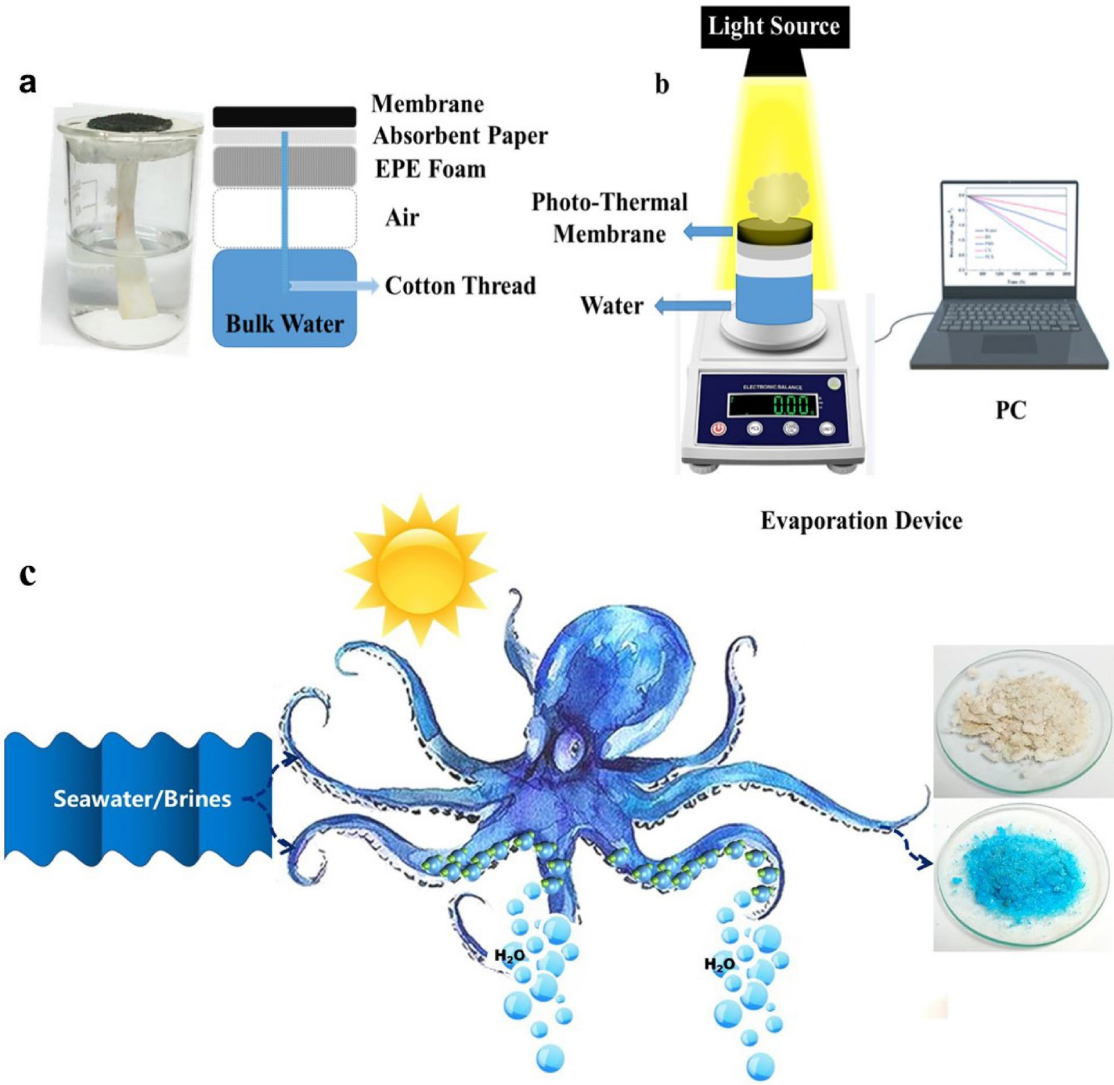
The proposed evaporation apparatus (**a**) Schematic of the full system integration. (**b**) Exploded view of the novel evaporation device, and graphical illustration of the dual function of the developed photothermal membrane (**c**).

The photothermal evaporation performance of the samples was evaluated under simulated sunlight conditions. A solar simulator (CEL-PE300E-3A, Au Light, China) provided the illumination, and an AM 1.5G filter was employed to mimic the spectral distribution of natural sunlight emitted by xenon lamps. The light intensity from the solar simulator was measured using a calibrated (Digital Light Meter − 200,000 Lux) and maintained at 1 kW m^−2^ (1 sun) throughout the experiments. The photothermal membrane was mounted onto a piece of ethylene propylene foam (EPR). A layer of absorbent paper was placed between the membrane and the foam, ensuring close contact with the PPy-coated side. This absorbent paper served as a water reservoir and delivery system. Water from a bulk reservoir was transported upwards through the hydrophilic channels within the EPE foam via a hydrophilic cotton thread, ultimately reaching the absorbent paper in contact with the photothermal membrane. The evaporation rate of water was quantified by monitoring the mass change of the solution using a high-precision electronic analytical balance (ML304T, METTLER-TOLEDO), as illustrated in Fig. 2a,b, in addition to graphical illustration of the freshwater generation and salt harvesting of the developed photothermal membrane (Fig. 2c).

To quantify the effectiveness of photothermal evaporation, the solar conversion efficiency for water evaporation (Ƞ) was evaluated as a key performance indicator, as defined in the following equation^28,29^:

1 $$\eta = \frac{{\left( {H_{v} + H_{s} } \right) \Delta V}}{Q}$$

where H_v_ is the heat of vaporization (2260 kJ kg^− 1^), H_s_ is the sensible heat of water, Δv is the improvement of water evaporation rate under illumination (kg m^− 2^ h^− 1^) (the difference in value on evaporation rates with and without light, Q (1 kW m^− 2^) is the intensity of simulated solar light for vertical irradiation^21^.

## Results and discussion

### Chemical vapor deposition polymerization (CVDP)

A PPy thin film was conformally deposited onto the substrates through in-situ CVDP. The CVDP was selected over conventional electro- or solution-phase polymerization because CVDP enables precise, solvent-free deposition of PPy as a conformal thin film, ensuring homogeneous coverage even on porous or textured substrates. This contrasts with solution-phase methods, where uneven wetting or agglomeration can occur. Unlike electrochemical polymerization (which requires acidic electrolytes and conductive substrates), CVDP avoids corrosive solvents. The resulting PPy layers exhibit enhanced adhesion and long-term stability under harsh conditions. CVDP operates at mild temperatures (≤ 100°C) with minimal waste, aligning with green chemistry principles.

**Scheme 1:**
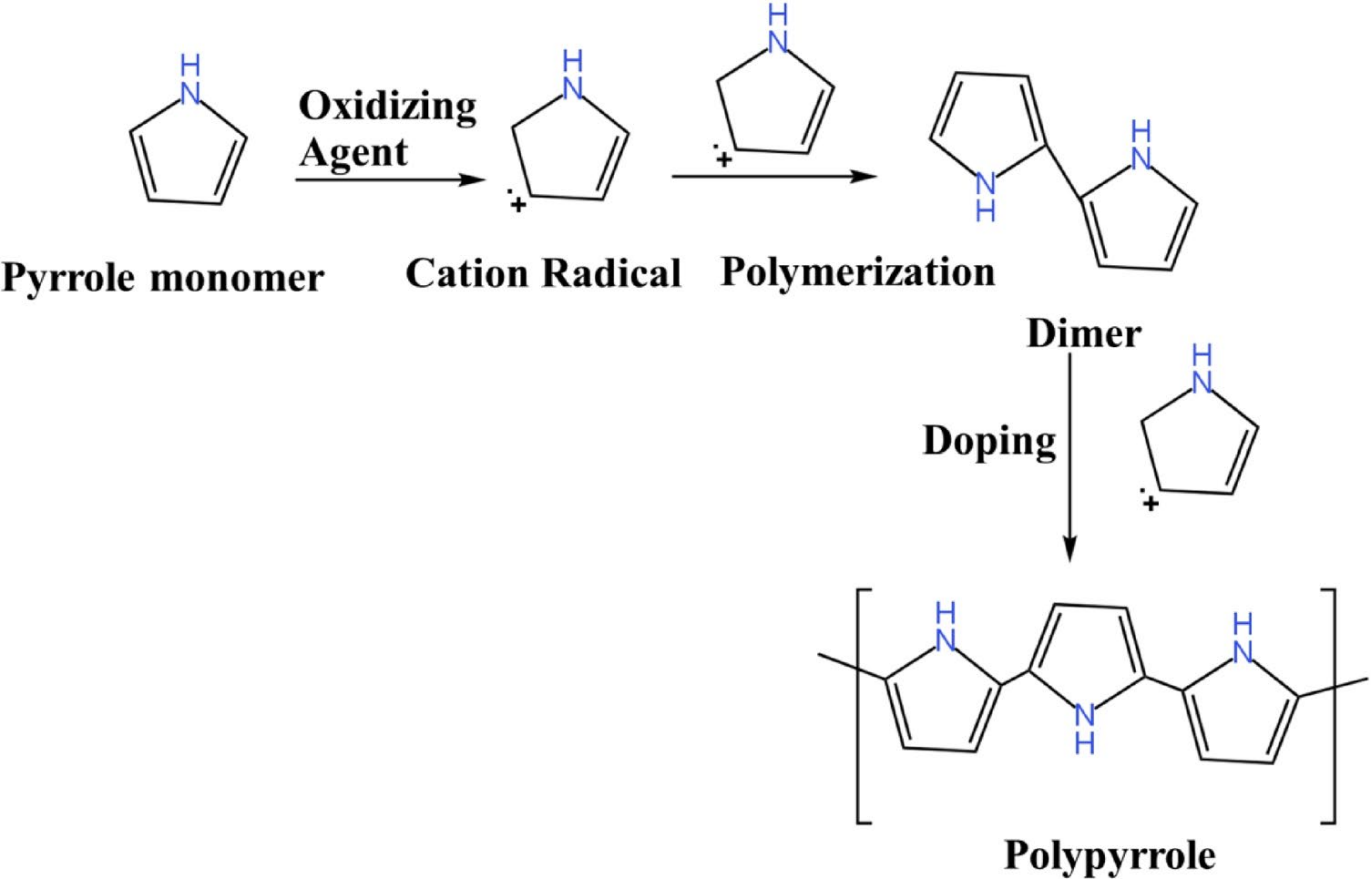
PPy polymerization via cation radical reaction mechanism.

The substrates obtained using various oxidizing agents acquired the color of the respective oxidizing agent solution, as shown in the supplementary file (Figures S1 to S4). At a specific temperature during the reaction, the volatility of the pyrrole monomer increases. This allows for better permeation of the pyrrole throughout the closed container. The oxidizing agent adsorbed on the substrate surface initiates the polymerization of pyrrole monomers. The reaction mechanism of the polymerization process is shown in Scheme 1. Following polymerization, a dark PPy thin film is deposited on the substrate surface using all oxidizing agents except Na_2_Cr_2_O_7_. In the case of Na_2_Cr_2_O_7_, a light brown layer is formed (Fig. S3 and S4). The influence of oxidizing agents on the initiation and efficiency of pyrrole polymerization can be investigated. This facile polymerization is likely attributable to the relatively high vapor pressure of the pyrrole monomer, which facilitates and enhances its participation in the reaction^30,31^. The simplicity of the CVDP enabled us to scale up pyrrole polymerization on a substrate with a surface area of 630 cm^2^ (equivalent to an A4 sheet) achieved using 0.4 mL of pyrrole monomer, as shown in Fig. 1b. The utilization of a small amount of pyrrole monomer represents a significant reduction compared to the quantities typically required in liquid-phase polymerization processes. In comparison to traditional liquid-phase methods, CVDP presents a scalable, cost-effective, and environmentally friendly alternative to traditional liquid-phase methods for the production of PPy-coated photothermal membranes. Its high efficacy is reflected in the minimal amount of pyrrole monomer required.

### Surface morphologies of the photothermal membranes

**Fig. 3:**
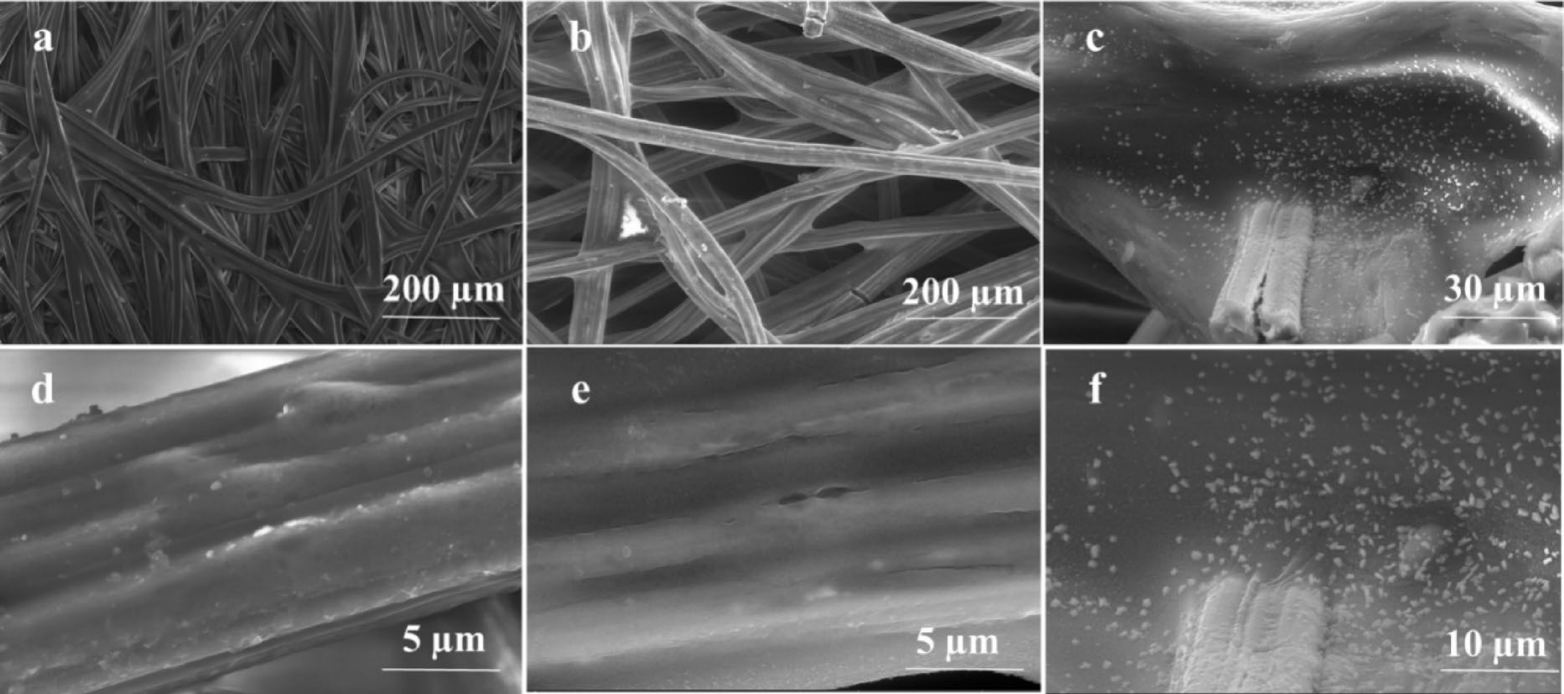
SEM images of (**a**, **d**) unmodified (Blank) substrate, and (**b**-**f**) modified non-woven polyester fabrics with 0.1 M CuCl_2_ and 10 μL PPy.

**Fig. 4:**
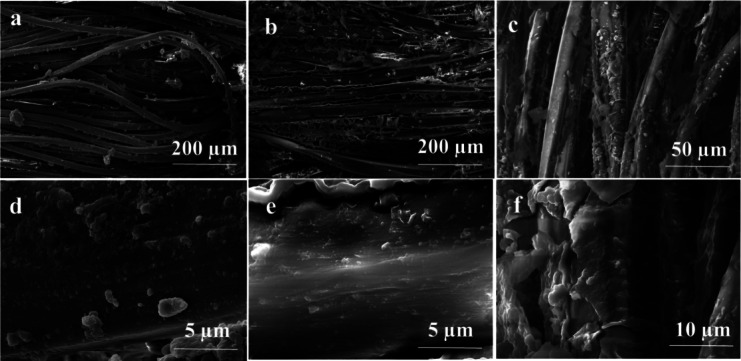
SEM of (**a**, **d**) unmodified (Blank) and (**b**-**f**) modified woven fabrics with 0.5 M APS and 20 μL PPy.

Scanning electron microscopy (SEM) was employed to characterize the surface morphology of the fabricated membranes (Figs. 3, 4). The polyester (non-wosven) substrate exhibited fine fibers with a smooth surface at high magnification (Fig. 3a,d). In contrast, the fabric (woven) substrate displayed thicker fibers (Fig. 4a,d). Following PPy deposition, the fibrous structure remained largely intact. However, the presence of small clusters and granules on the fiber surfaces indicated a modification of the surface microstructure. This suggests successful PPy deposition and its impact on the surface topography. Furthermore, a distinct PPy coating layer was observed covering the substrate skeleton, as evident in the fractured surface. This uniform and nanoscale-thick PPy coating transformed the initially smooth surface into a rougher one. Previous studies have demonstrated that such rough surfaces with abundant micro-structures can significantly enhance light harvesting efficiency^33^. SEM images reveal that the pristine commercial fabric substrates—both woven and non-woven—already exhibit numerous surface particles. These particles are likely attributed to fiber agglomeration, residual dust, or incomplete removal of manufacturing residues due to insufficient washing during the fiber production process.

### Structural composition of the photothermal membranes

To evaluate the successful deposition of the oxidizing agent on substrates and ensure the structure of the oxidizing agent, XRD spectra of different oxidizing agents were obtained. XRD pattern of the nonwoven fabric exhibits peak at 2θ = 12.5°, 17.1°, 20.6°, 21.9°, 25.5°, 29.6° and 31.8°^34,35^. The characteristic peak at 2θ = 17.2° represents iron (II) chloride tetrahydrate (secondary phases)^36^. Peak at 2θ = 21.9° and 31.8° represent iron (III) chloride hydrate (0.1M, FeCl_3_). Peak at 2θ = 12.3°, 17.0°, 22.0°and 25.4° represent ammonium persulfate^37^, peak at 2θ = 20.1° represents ammonium sulfate (secondary phases) (0.1M, APS). Peaks at 2θ = 22.1°, 29.0°, 34.2°, 35.0°, 41.0°, 44.9° and 57.5° represents copper chloride hydrate^38^ (0.1M, CuCl_2_). Peak at 2θ = 17.2° represents potassium manganese oxide, peak at 2θ = 37.3° represents manganese hydroxide (secondary phases), and peak at 2θ = 66.1° represents potassium manganese oxide hydrate (secondary phases) (0.1M, KMnO_4_). Peak at 2θ = 17.2°, 22.0°, 29.8°, and 31.8° represents sodium chromium oxide hydrate (0.5M, Na_2_Cr_2_O_7_).

**Fig. 5:**
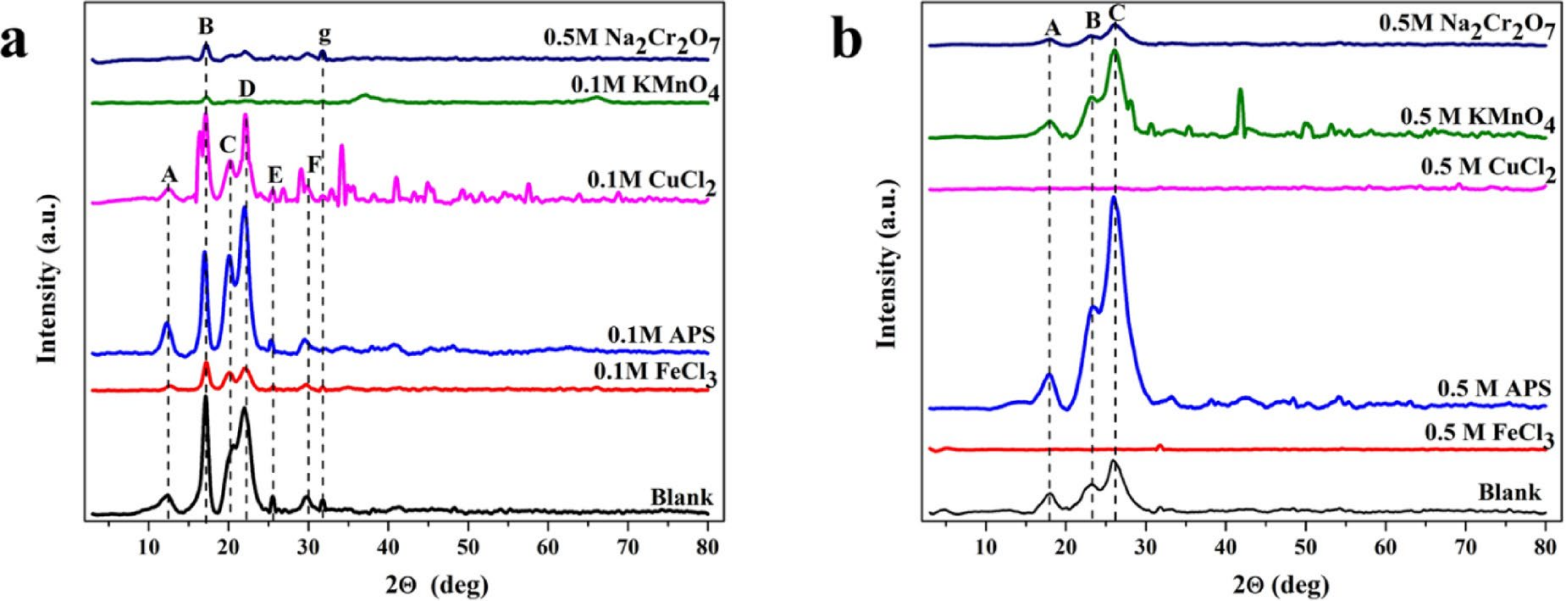
XRD patterns of (**a**) non-woven and (**b**) woven polyester fabrics before and after exposure to oxidizing agents.

Secondly, XRD spectra of different oxidizing agents on fabric (woven) substrate are shown in Fig. 5b. XRD pattern of fabric (woven) exhibits peaks at 2θ = 18.1°, 23.3°, 26.0°, and 31.7°. The characteristic peak at 2θ = 31.7° represents iron chlorate hydrate (0.5M, FeCl_3_). Peaks at 2θ = 18.0°, 25.8°, and 48.4° represent ammonium sulfate oxide (secondary phases)^39^ (0.5M, APS). Peaks at 2θ = 49.8°, 57.9°, 69.2° and 73.3° represent copper chloride hydrate^40^ (0.5M, CuCl_2_). Peaks at 2θ = 19.5°, 22.8°, 25.0°, 25.8°, 27.7°, 30.2°, 34.9°, 41.0°, 41.4°, 47.3°, 47.9°, 49.4°, 50.0°, 51.7°, 52.7°, 64.6°, 66.3°, 72.2°, 76.1° and 76.6° represent potassium manganese oxide (0.5M, KMnO_4_)^41,42^. Peaks at 2θ = 17.8° and 26.1° represents sodium chromium oxide hydrate (0.5M, Na_2_Cr_2_O_7_)^28^.

Sticks shown in the XRD pattern represent the common peak that appeared in all samples, referring to the characteristic peak of blank samples, but some of these peaks disappears in some sample. In addition, some peak intensities become very weak because of the higher crystallinity of the oxidizing agent on the sample surface. The XRD results confirm the successful precipitation of different oxidizing agents on the surface of the substrate to initiate the polymerization process. The appearance of secondary phases is attributed to the oxidative degradation or decomposition of the oxidizing agent on the surface^]^.

As evidenced in Fig. 5, the diffraction patterns of the woven and non-woven fabrics differ significantly. The woven fabric is characterized by shorter, broader peaks, suggesting a lower degree of crystallinity or smaller crystallite size. In contrast, the non-woven fabric displays high, sharp peaks with significant changes, indicative of a more ordered crystalline structure.

### Investigation of solar light absorption

**Fig. 6:**
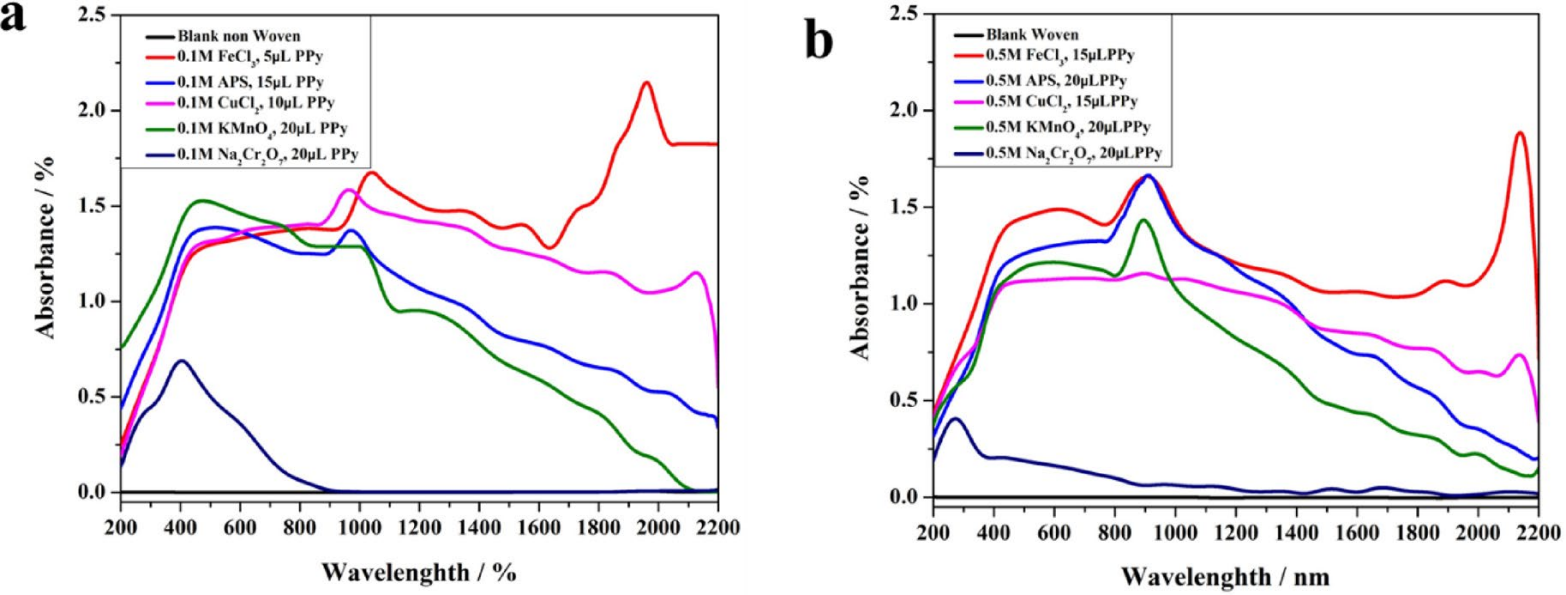
Light absorbance spectra demonstrating the effect of polypyrrole (PPy) deposition on (**a**) nonwoven and (**b**) woven substrates.

To evaluate the light-harvesting ability, the light absorption properties of the deposited PPy layers were investigated with a UV–Vis–NIR spectrophotometer (Fig. 6).

The employment of sodium dichromate as an oxidizing agent during membrane fabrication resulted in exceptionally high levels of light absorption. This phenomenon is attributed to the inadequacy of sodium dichromate in facilitating complete pyrrole polymerization on the substrate, readily discernible by the naked eye. In successful polymer formation, the substrate exhibits a deep black color. Conversely, the use of sodium dichromate yields a light brown hue. In contrast, the utilization of iron chloride, copper chloride, ammonium persulfate, and potassium permanganate at concentrations of 0.1 M and 0.5 M, respectively, led to a progressive decrease in both diffuse reflectance and transmittance across the wavelength spectrum of 200–2200 nm. Notably, the treated substrates with iron chloride and copper chloride as the oxidizing agents in non-woven substrate displayed the lowest values of diffuse reflectance and transmittance, falling below 4%. Through optimization, PPy-coated samples achieve remarkable light absorption capabilities, exceeding 94% on average across the broad solar spectrum from 200 to 2500 nm. This high efficiency is attributed to the negligible transmittance observed in these optimized samples.

The non-woven substrate’s inherent properties of high diffuse reflectance and transmittance resulted in insufficient light absorption, rendering it inadequate for light-absorbing applications. The remarkable light-harvesting efficiency observed in the modified samples can be primarily attributed to the PPy coating layer for two key reasons. Firstly, the dark color of photothermal materials arises from their broadband light absorption across the solar spectrum (UV–VIS-NIR). This property enables efficient light-to-heat conversion. Secondly, the deposition of PPy promotes the formation of numerous microstructures on the surface. Rough surface microstructures lead to effectively reducing the material’s diffuse reflectance.

### Surface temperature measurements

**Fig. 7:**
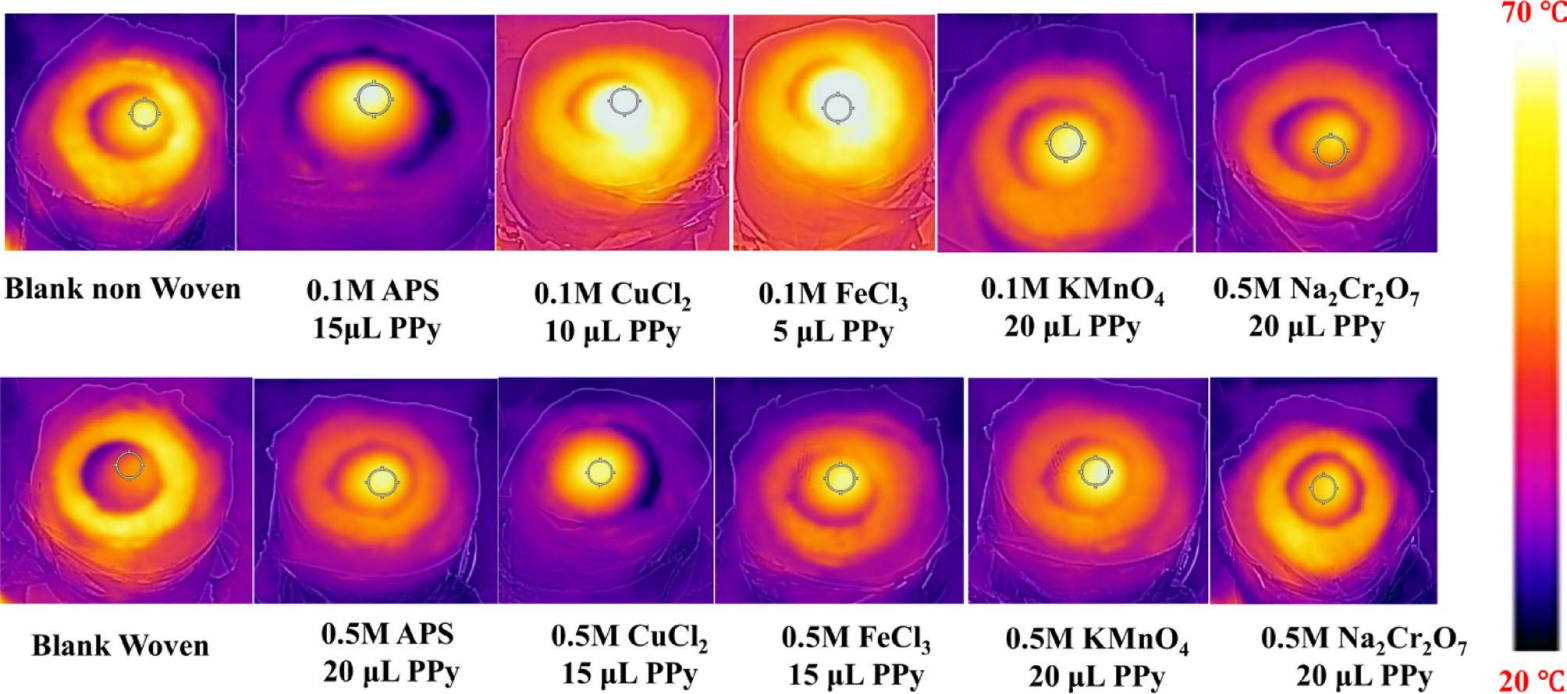
Surface temperature of the photo-thermal membranes after illumination for 30 min at 1 sun.

The steady-state surface temperatures of the developed photothermal membranes were measured using infrared (IR) imaging under simulated sunlight with an intensity of 1 kW m^− 2^, equivalent to 1 sun, as shown in Fig. 7. The blank samples of non-woven fabric exhibited a higher equilibrium temperature (37°C) compared to the woven fabric (34°C) after 30 min of simulated sunlight irradiation. Following PPy deposition via CVDP, the modified substrates exhibited a gradual temperature increase when treated with efficient oxidizing agents such as ferric chloride, ammonium persulfate, and copper chloride. The surface temperature of good performing membranes fabricated with 0.1 M CuCl_2_ and 10 µL PPy (non-woven substrate) and 0.5 M APS and 20 µL PPy (woven substrate) reached up to 65 ± 2.7°C and 60 ± 2.1°C, respectively. This reasonably explains the higher evaporation rate of these photothermal membranes. The higher surface temperatures of the PPy-coated membranes can be attributed to their improved light-harvesting efficiency, which is consistent with the previously discussed results. This suggests that the changes in surface morphology caused by the PPy deposition and oxidation treatment enhances the ability of the membranes to capture and convert sunlight into heat, leading to the observed temperature rise.

### Contact angle measurements

**Fig. 8:**
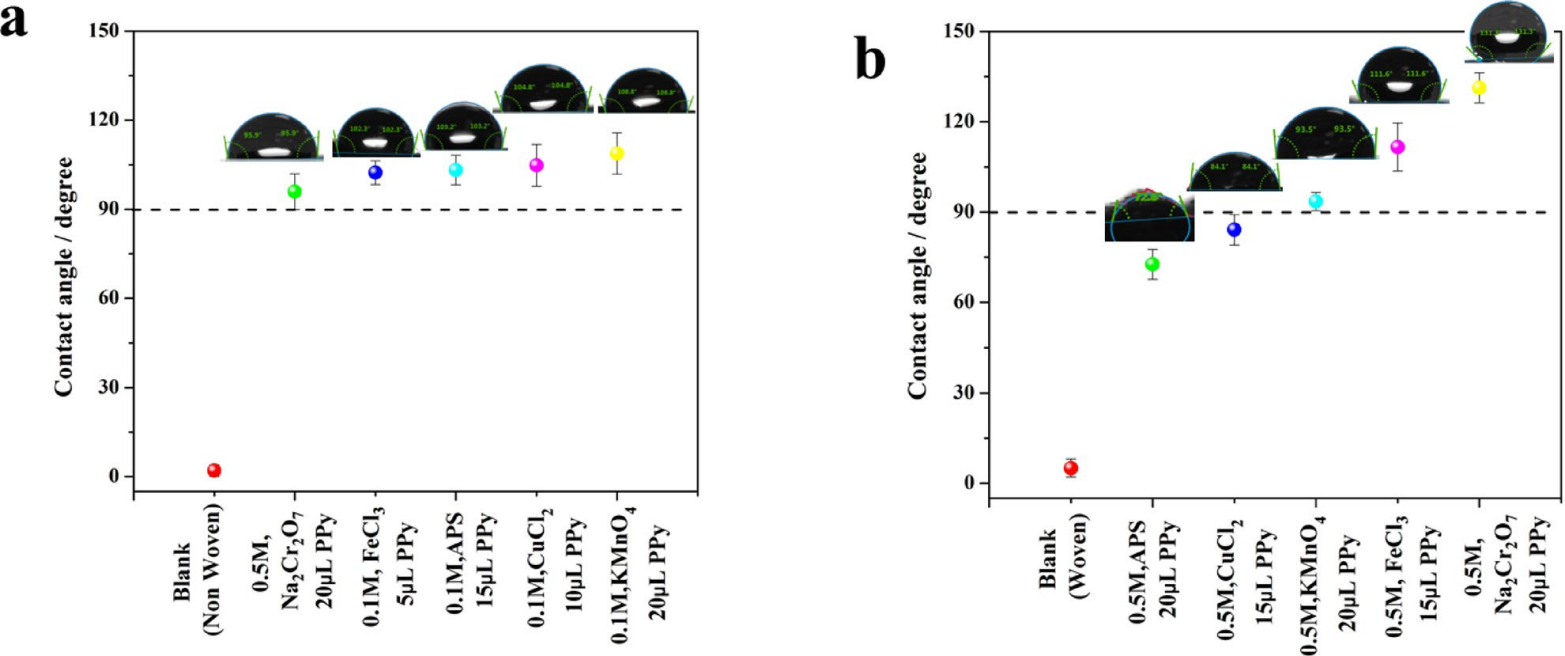
Water contact angles of photo-thermal membranes fabricated on (**a**) non-woven and (**b**) woven substrates, treated with different oxidizing agents.

Beyond photothermal conversion efficiency, achieving optimal water transport is equally crucial for maximizing solar-driven evaporation. In conjunction with efficient light absorption, effective water management through the material is paramount for achieving high rates of solar-driven evaporation. A wettable surface, measured by water contact angle, can contribute to fast water transport. Figure 8 shows the contact angles of membrane samples with PPy deposition compared to the blank. The deposited PPy layers using non-woven fabric as a substrate (Fig. 8a) showed a highly hydrophilic surface with a contact angle of less than 4°. The nonwoven blank substrate exhibited exceptional hydrophilicity, causing water droplets to readily spread upon contact after deposition of the PPy layer, resulting in a gradual increase in the top surface contact angles for membranes fabricated with varying oxidizing agents. The contact angles of 0.5 M Na_2_Cr_2_O_7_ with 20 µL of pyrrole, 0.1 M FeCl_3_ with 5 µL of pyrrole, 0.1 M APS with 15 µL of pyrrole, 0.1 M CuCl_2_ with 10 µL of pyrrole, and 0.1 M KMnO_4_ with 20 µL of pyrrole were up to 95.9°, 102.3°, 103.2°, 104.8°, and 108.8°, respectively. The observed persistence of water spreading on the PPy-coated polyester membranes can be attributed to two mechanisms. First, the inherent capillary action arises from the porous structure of the fabric substrates, and secondly, the presence of the deposited hydrophobic PPy layer.

The developed photothermal membranes using woven fabric substrate with different oxidizing agents showed a contact angle of 131.3°, 111.6°, 72.6°, 84.1° and 93.5° using 0.5 M Na_2_Cr_2_O_7_ with 20 µL of pyrrole, 0.1 M FeCl_3_ with 5 µL of pyrrole, 0.1 M APS with 15 µL of pyrrole, 0.1 M CuCl_2_ 10 µL of pyrrole, and 0.1 M KMnO_4_ 20 µL of pyrrole, respectively.

### Functional composition of the photothermal membranes

**Fig. 9:**
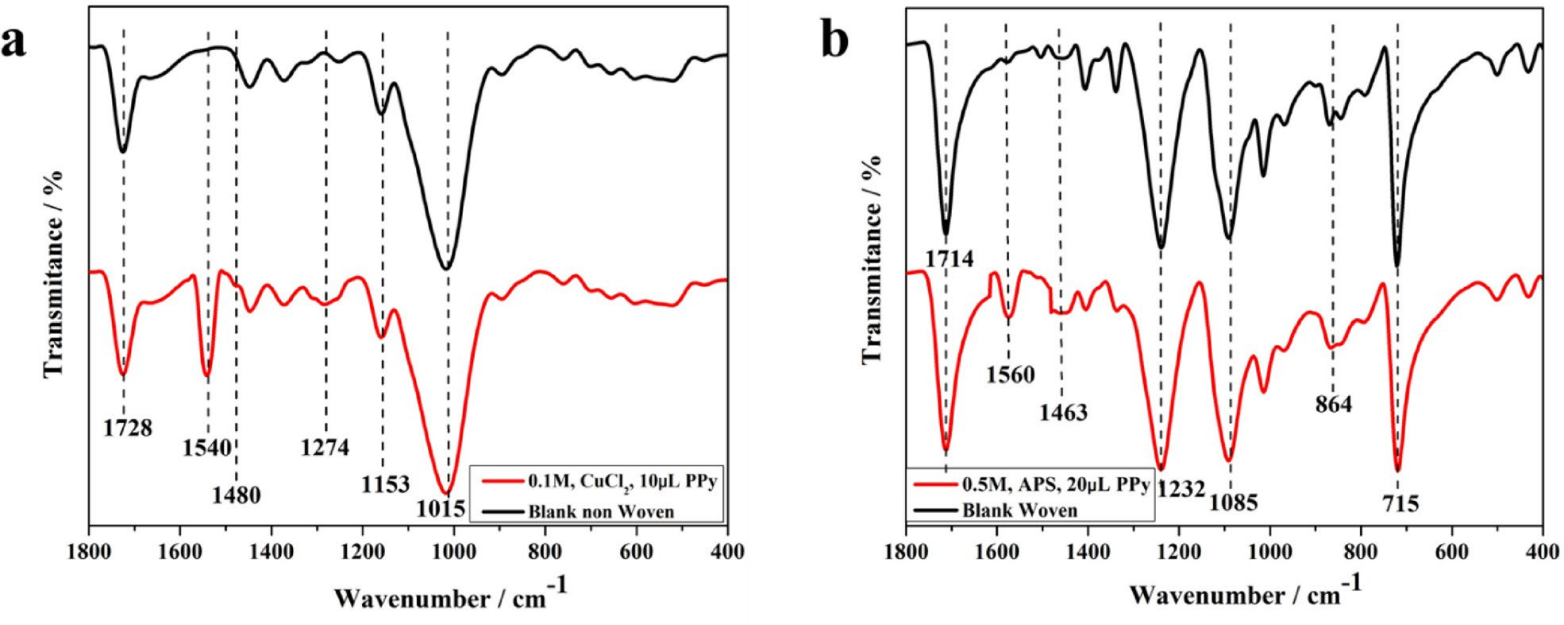
FTIR analysis of the developed photo-thermal membranes, FTIR spectra confirm the successful functionalization of both (**a**) non-woven and (**b**) woven fabric substrates.

In order to understand the chemical basis for the observed unusual wettability, FTIR spectroscopy was employed, as shown in Fig. 9. The FTIR spectra of non-woven fabric (polyester) showed characteristic peaks at 1015, 1153 and 1728 cm^− 1^ representing C–O stretching of alkyl group, C–O stretching of ester group, C=O stretching and OH stretching, respectively. The woven fabric showed characteristic peaks at 715, 864, 1085, 1232 and 1714 cm^− 1^ representing C=C, CH bending, C–O of ether group, and C=O group, respectively. After PPy deposition on the two substrates (woven and non-woven), new peaks appeared at 1274, 1480 and 1540 cm^− 1^ of non-woven fabric, representing C–N stretching vibration, vibration of the pyrrole ring, while the woven fabric showed peaks at 1463 and 1560 cm^− 1^ representing C–C/C=C stretching, respectively^]^.

### Surface roughness investigation

**Fig. 10:**
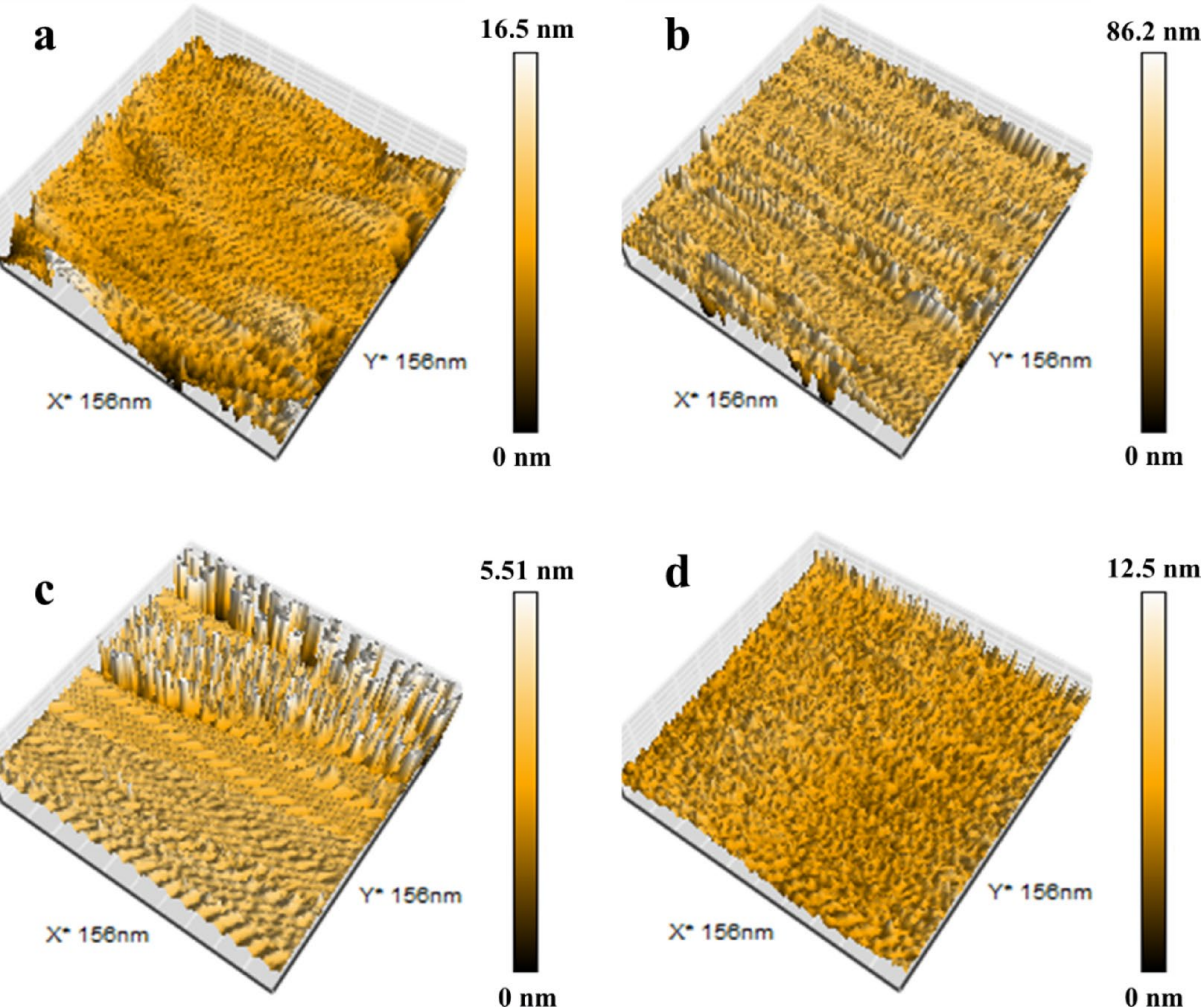
AFM images of the non-woven fabric before and after deposition of PPy (**a**, **b**), and the woven fabric before and after deposition of PPy (**c** and **d**).

To facilitate a comparative analysis of surface morphology, the mean roughness (Ra) was quantified. This parameter represents the arithmetic mean of the absolute deviations of the roughness profile from the mean line. This parameter serves as an effective descriptor for the topographic variations across the surface^47^. For example, the roughness of non-woven and woven fabric (blank) was 5.18 nm and 12.37 nm, respectively, as shown in Fig. 10. After deposition of the PPy layer roughness of the membrane increased to 10.77 nm and 43.22 nm for non-woven and woven fabric, respectively. Thus, the roughness of the photothermal membranes is 2.1 and 3.5 times higher compared to non-woven and woven fabric (blank), respectively. It can therefore be concluded that the application of the PPy coating significantly enhances surface roughness by introducing a textured layer.

Furthermore, the porosity of the woven and non-woven fabric with and without PPy deposition was determined, as shown in Fig. S5. A comparative analysis shows that PPy deposition significantly reduces membrane porosity from 78.6% and 76.7% to 62.3% and 60.7% for non-woven and woven fabrics, respectively. This reduction is attributed to the hydrophobic PPy layer, which alters the membrane microstructure and decreases pore accessibility.

### Performance evaluation of the developed photothermal membranes

#### Evaluating the evaporation rate under 1 sun

**Fig. 11:**
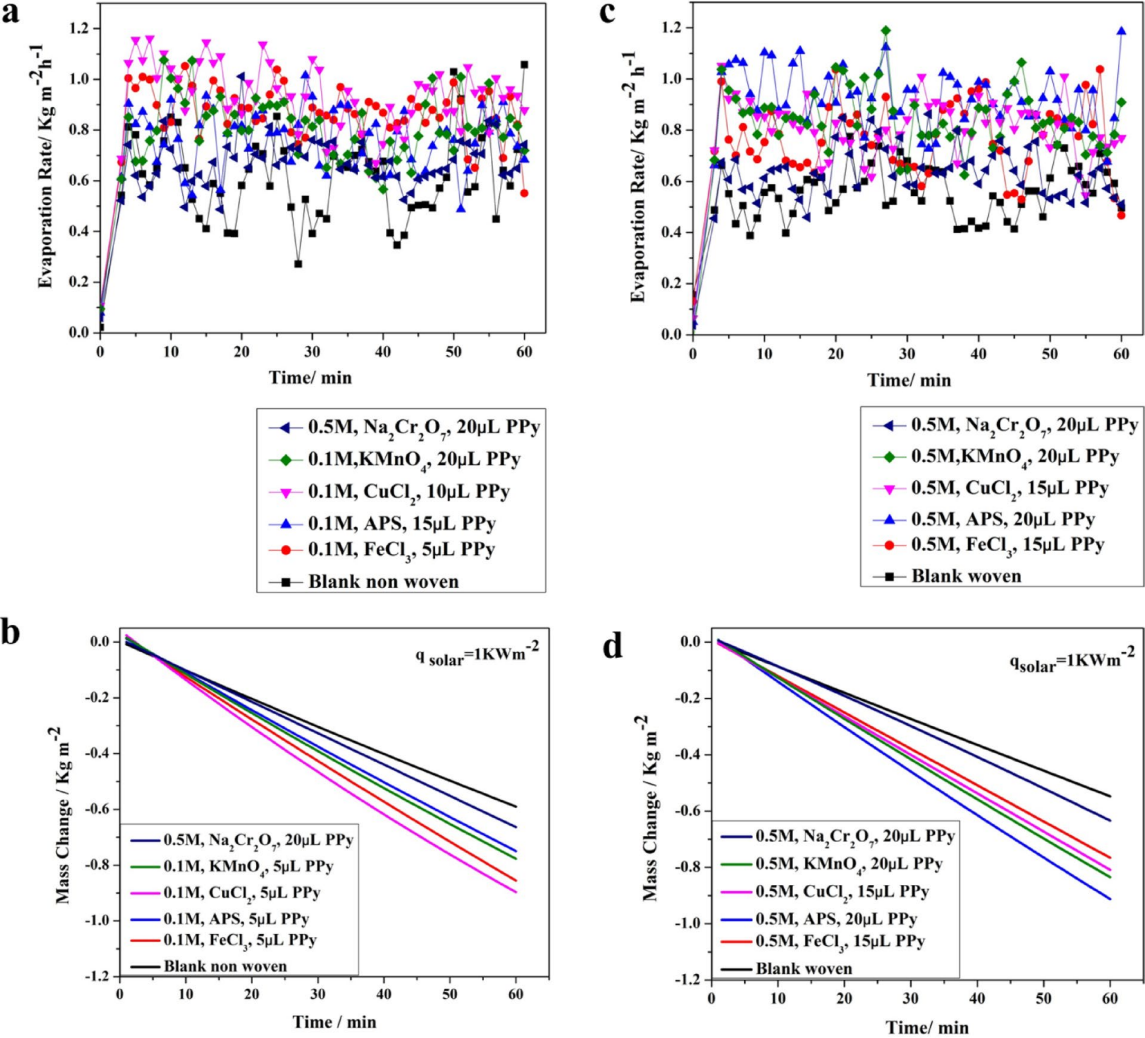
Evaporation rate and mass change of the developed PPy layer on non-woven (**a**, **b**) and woven fabrics (**c**, **d**).

The determined evaporation rates reveal that the blank non-woven and woven fabrics were 0.62 kg m^− 2^ h^− 1^, and 0.54 kg m^− 2^ h^− 1^ which demonstrated a roughly 300% increase in evaporation rate compared to water sample without any photothermal membranes (0.22 kg m^− 2^ h^− 1^), as shown in Fig. 11. Following the deposition of PPy onto the non-woven and woven fabric substrates, a significant enhancement in water evaporation rate was observed. The non-woven photothermal membrane with both oxidizing agents of CuCl_2_ and FeCl_3_ (0.1 M) showed the excellent evaporation rates of about 0.95 and 0.88 kg m^− 2^ h^− 1^, respectively. Comparable results were obtained using the woven photothermal membrane using APS and KMnO_4_ (0.1 M) as oxidizing agents that showed evaporation rates of 0.93, and 0.85 kg m^− 2^ h^− 1^, respectively. As a conclusion, the deposited PPy layer enhanced the evaporation rate with an average improvement of 1.5 times, and 1.6 times higher than the blank for the non-woven and woven photothermal membranes, respectively.

To validate the efficacy of PPy as an efficient coating for the photothermal membrane, the same membrane was tested with a black ink dye coating (Fig. S6, supplementary file) of the woven and non-woven fabrics. A comparison of the results demonstrated that PPy is considerably more effective than black dyes. As illustrated in Fig. S6a, the evaporation rate after coating did not improve significantly compared to its prior state: it increased from 0.62 and 0.54 to 0.66 and 0.554 for non-woven and woven membranes, respectively. Similarly, Fig. S6b indicates that the solar thermal conversion efficiency also exhibited minimal improvement, rising from 36 and 32% to 38% and 32.2% for non-woven and woven membrane, respectively.

#### Solar thermal conversion efficiency

**Fig. 12:**
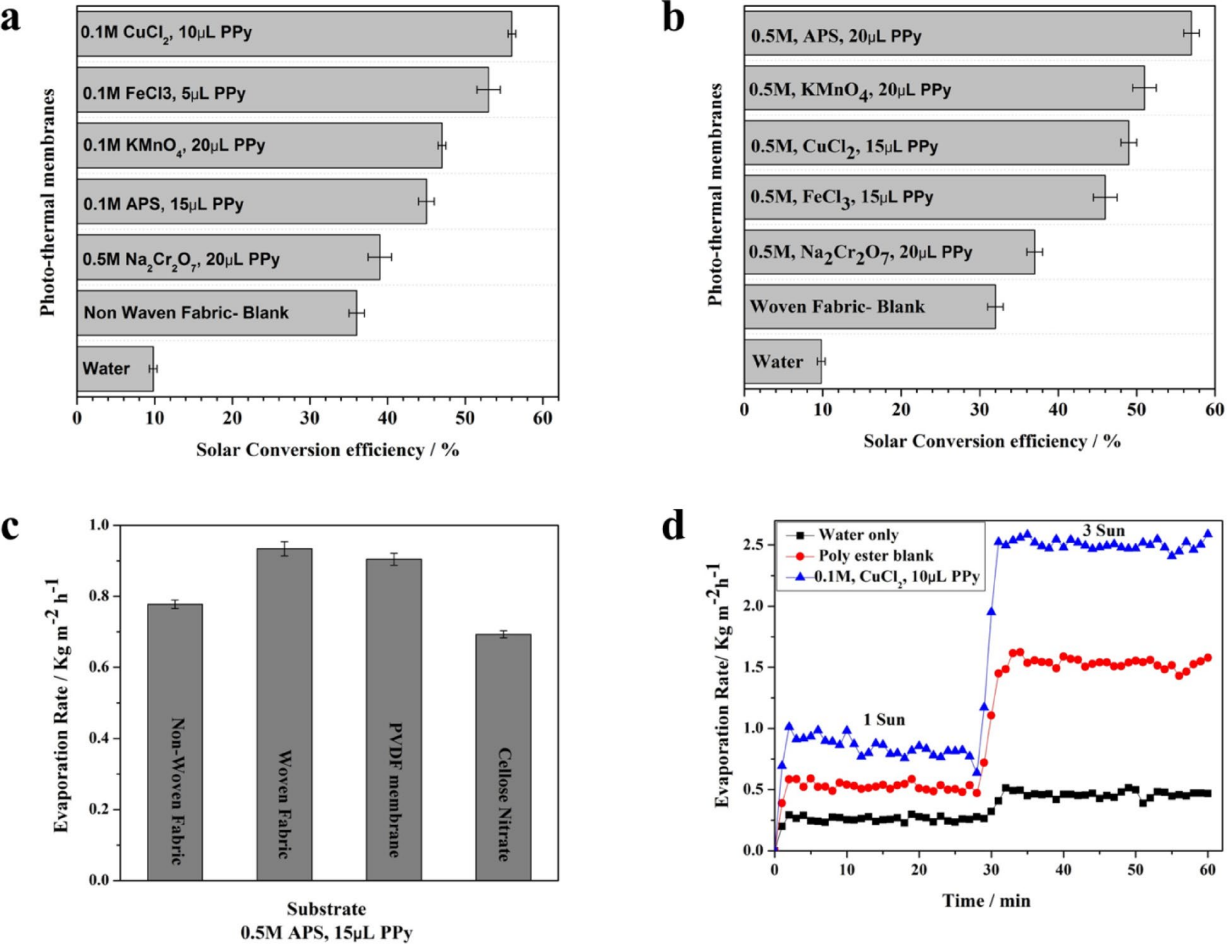
Solar thermal conversion efficiency of the developed photothermal membranes using the non-woven fabrics (**a**) and woven fabrics (**b**) with different oxidizing agents. (**c**) The average evaporation rate of the photo-thermal membranes using different substrates with the same oxidizing agent (0.5M APS) and the same amount of pyrrole (15 µL). (**d**) The evaporation rate of the nonwoven fabric (polyester) with and without PPy layer under one and three sun illumination, compared with water.

The maximum obtained solar conversion efficiency for the developed photothermal membranes with a deposited layer of PPy on the woven and non-woven fabrics showed about 57%, as shown in Fig. 12a,b. It is noted that solar thermal conversion efficiency is relatively low, due to the use of a very small amount of pyrrole (5–20 µL). Small amounts of pyrrole were used to measure the efficiency of the oxidizing agents developed in this work to start the polymerization process and form the polymer layer on the surface of the substrate. With the smallest amount, and for this small amount of monomer used, the equivalent evaporation rate for these membranes is considered excellent. The observed high evaporation rate is due to the following three aspects; first, to the efficiency of the polymerization process, a highly effective oxidizing agent might ensure complete activation of pyrrole monomers, leading to a well-formed and functional polymer layer on the substrate surface, second, chemical vapor deposition polymerization (CVDP) likely generated PPy particles or small clusters on the membrane surface.

These surface microstructures are believed to be responsible for a reduction in diffuse reflection, thereby enhancing light harvesting efficiency. Third, the favorable wettability of PPy-coated substrates (non-woven and woven fabrics) likely facilitates water transport to the membrane surface, promoting efficient evaporation. PPy is a well-established conductive polymer known for its straightforward synthesis and remarkable environmental stability. These properties make it a promising candidate for applications in solar thermal conversion^48,49^.

While the fabricated PPy-coated membrane demonstrated a viable solar evaporation rate, its calculated solar-to-thermal conversion efficiency of 57% is acknowledged to be below the highest values reported in the literature for specialized photothermal materials. This performance can be attributed to several key factors; the substrate was chosen with excellent mechanical stability, but this leads to higher conductive heat losses compared to highly insulating substrates due to the macroporous structure of the fabric. The porosity of the PPy layer is crucial for water transport, but leading to more parasitic heat loss downwards.

PPy exhibits excellent versatility in adhering to various substrates through both electrochemical polymerization and chemical oxidation polymerization techniques. This versatility allows for its facile formation of electrodes and energy storage devices on diverse materials^]^. Motivated by the versatility of PPy for adherent coatings, this study explores the deposition of PPy layers on various substrates using a moderate CVDP technique. The average evaporation rate of different substrates with the same oxidizing agent (APS) and the same amount of pyrrole used to develop the photothermal layer was determined and presented as shown in Fig. 12c. Fabric substrates exhibited excellent composite formation with PPy. The PPy deposited layer displayed robust mechanical resilience during preparation and testing. The determined average evaporation rate under 1 sun illumination of woven fabric was 0.93 kg m^− 2^ h^− 1^, for non-woven fabric was 0.77 kg m^− 2^ h^− 1^ for PVDF was 0.90 kg m^− 2^ h^− 1^ and for cellulose nitrate was 0.693 kg m^− 2^ h^− 1^, as shown in Fig. 12c.

Our findings suggest that under identical polymerization conditions with the same oxidizing agent, the evaporation rate of photothermal membranes varies across different substrates. This disparity likely arises from two key factors: the efficiency of polymer layer formation on the substrate surface, and the wettability characteristics of the substrate. Material selection plays a critical role in ensuring substrate performance across varying water chemistries. For instance, fabrics (woven) exhibit susceptibility to corrosion in acidic environments, while, PVDF substrates demonstrate a propensity for gradual degradation in alkaline solutions. A comparison of different fabricated photothermal membranes was included in Table S1 (Supplementary file).

#### Evaluating the evaporation rate under 3 suns

Given the superior light absorption capacity of PPy, the PPy-modified fabric membranes (woven and non-woven) are anticipated to exhibit excellent photothermal properties when subjected to irradiances of 1 and 3 suns, respectively. Accelerated observation and measurement of photothermal effects can be achieved by exposing the material to a light intensity thrice that of solar irradiance. This facilitates rapid assessment of a material’s suitability for concentrated solar radiation environments. Furthermore, the cyclic temperature variations induced by alternating illumination at 1 and 3 suns for five cycles confirmed the robust photo-stability of the PPy-modified membranes^52^. While a solar concentration of three times terrestrial levels might seem extreme, it is essential to recognize that many real-world applications utilize concentrated sunlight. Solar concentrators, such as parabolic mirrors or lenses, can focus incident solar radiation onto a small region, producing irradiance levels significantly greater than one solar constant. By subjecting materials to a solar concentration of 3 suns, we can more accurately replicate high-intensity conditions and evaluate their practical utility. Increasing irradiance levels can aid in identifying a material’s performance limits. By subjecting the material to extreme conditions, we can elucidate its stability, degradation mechanisms, and avenues for enhancement. This knowledge is essential for the development of highly efficient and durable photothermal materials. For comparative purposes, the intrinsic evaporation rate of pure water under dark conditions at 25°C is measured as 0.075 kg m^− 2^h^− 1^ ^25,53^. Consequently, the mean water evaporation rates for the 0.1M CuCl_2_-treated non-woven fabric with a PPy coating of 10 µL are determined to be 0.93 and 2.91 kg m^− 2^ h^− 1^ under solar irradiances of 1 and 3 suns, respectively. Figure 12d illustrates the comparison of the evaporation rate between water, PVDF blank, polyester (non-woven fabric) without and with deposited PPy layer using 0.1M CuCl_2_, as the oxidizing agent and 10 µL pyrrole under 1 and 3 suns illumination.

#### Simultaneous salts extraction and freshwater harvesting

**Fig. 13:**
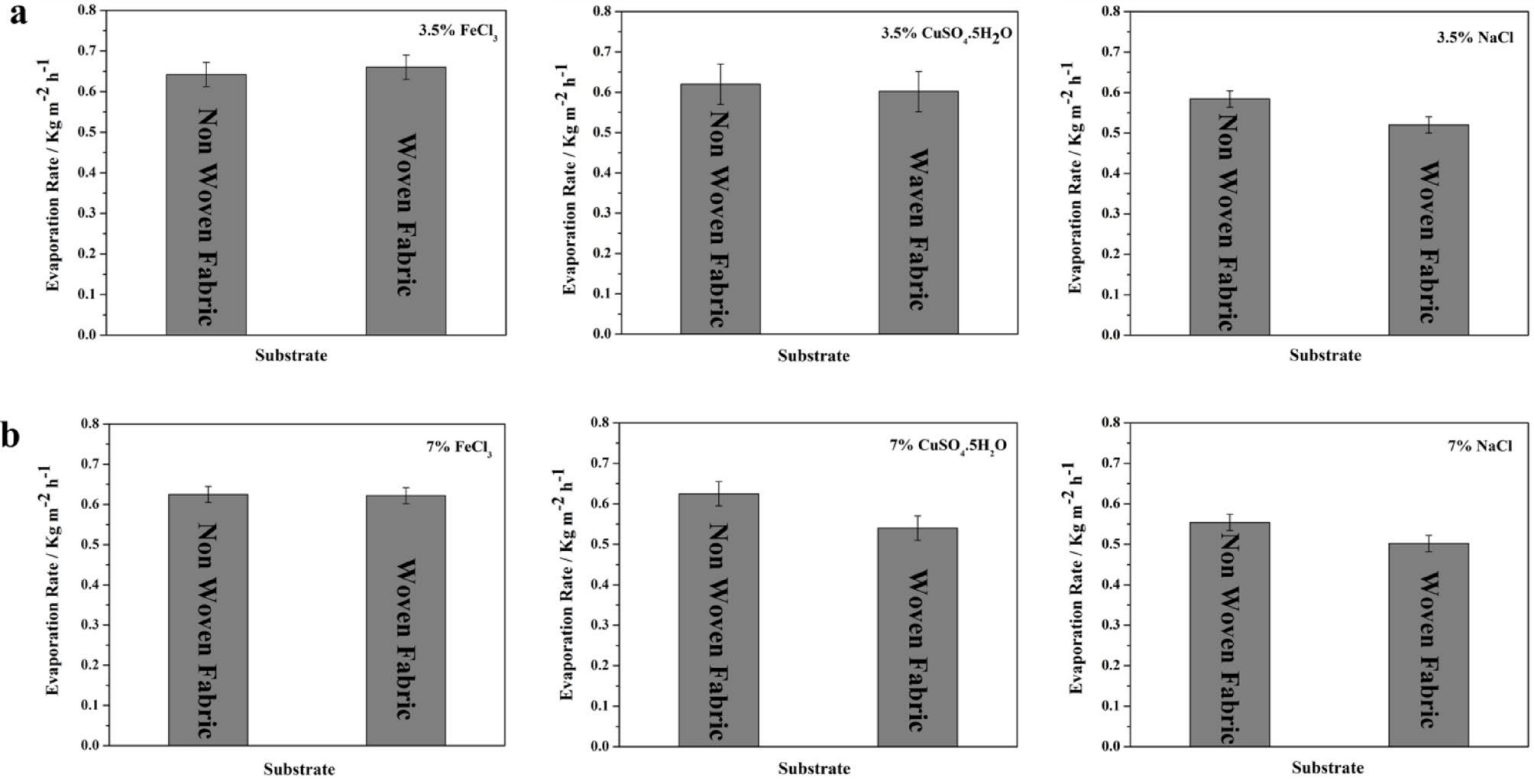
The average evaporation rates of non-woven (0.1M FeCl_3_, 5 µL pyrrole) and woven fabric (0.5M FeCl_3_, 15 µL pyrrole) photothermal membranes of three different solutions (FeCl_3_, CuSO_4_.5H_2_O, and NaCl) at low and high concentrations (**a**) 3.5% and (**b**) 7.0%.

The photothermal membrane facilitates the absorption of solar energy and its subsequent conversion into thermal energy. Consequently, the water layer permeating the membrane experiences a temperature increase, triggering evaporation. The generated vapor was then condensed and collected, yielding purified water for utilization. The applicability of these membranes extends beyond solar-driven water purification. They can be utilized for salt extraction from solutions or for concentrating dilute salt solutions while simultaneous harvesting freshwater. This functionality is achieved by leveraging the membrane’s ability to facilitate solar energy absorption and conversion into heat. The non-woven and woven fabric photothermal membranes were tested against different saline solutions at low and high concentrations as shown in Fig. 13. The type of saline solutions, the concentration and solution viscosity are crucial parameters in determining evaporation efficiency and membrane fouling. A high concentration (high viscosity) solution contributes to a slight decrease in the evaporation rate compared to the same saline solution at a lower concentration^54,55^.

**Fig. 14:**
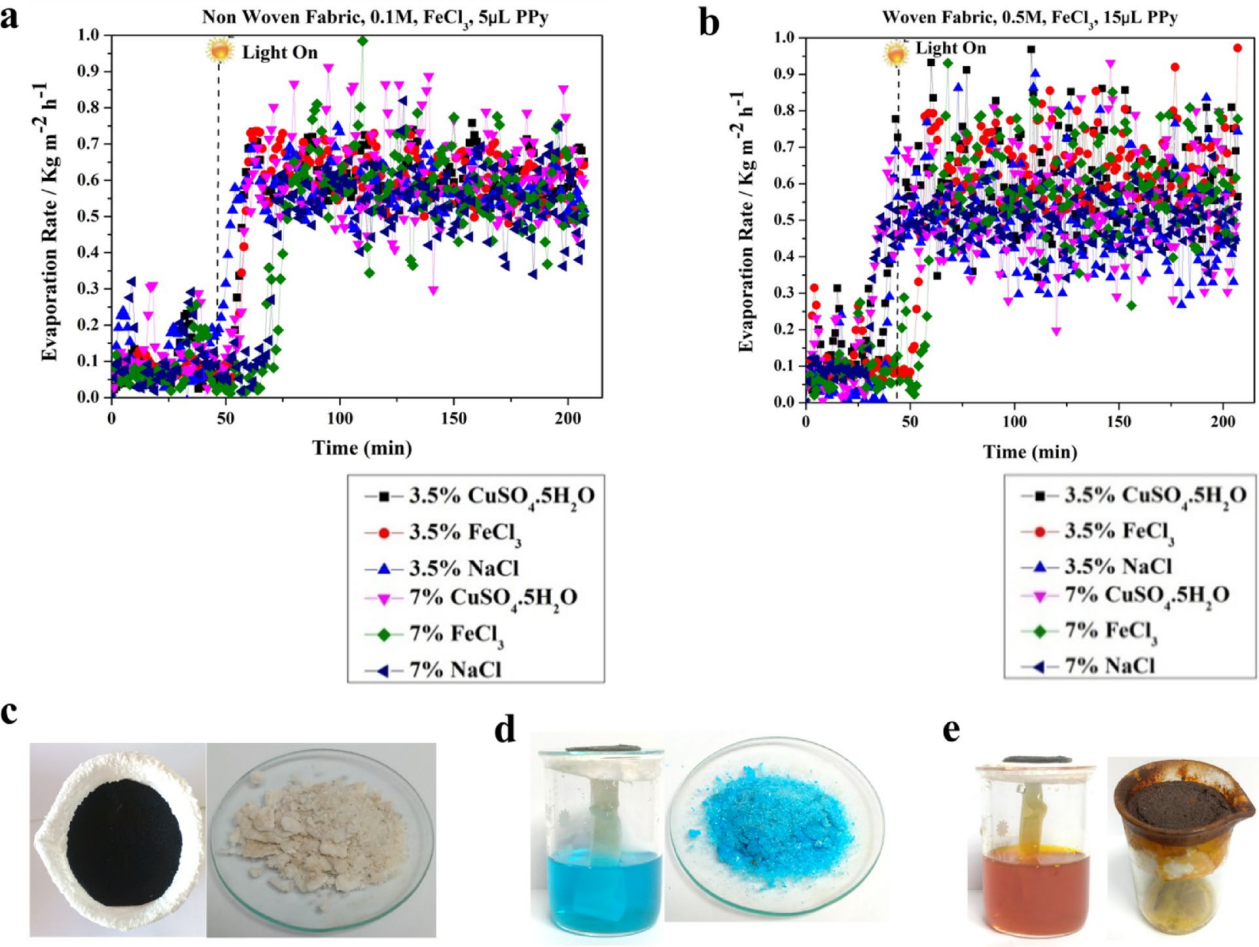
The evaporation rate of different salts solutions at 1 sun using (**a**) non-woven and (**b**) woven fabric photothermal membrane at different conditions (**a**) 0.1M FeCl_3_ as oxidizing agent and 5 µL pyrrole, and (**b**) 0.5M FeCl_3_ as oxidizing agent and 15 µL pyrrole.

The resulting temperature increase promotes the evaporation of water from the solution, consequently concentrating the desired salts. Membranes with pores larger than the hydrated salt ions allow for salt extraction, effectively removing the salts from the solution. Conversely, membranes with pores smaller than the hydrated salt ions permit the passage of water molecules, leading to concentration of the salts in the remaining solution. This separation mechanism is primarily driven by a size-exclusion effect. The described scenario involves extracting salts through evaporation using innovative photothermal membranes. The water evaporates, leaving behind accumulated salts on the container’s edge and membrane sides, with capabilities of self-salt repelling and avoiding salts accumulation on the photothermal membranes with no fouling or pore blocking. Three different solutions were studied for simultaneous freshwater and salts extraction using PPy-coated nonwoven fabric photothermal membrane with 3.5 and 7.0% of NaCl, FeCl_3_, and CuSO_4_.5H_2_O, as shown in Fig. 14. These solutions were tested under 1 sun to determine the precise evaporation rate followed by natural evaporation in ambient for a few days to reach the complete evaporation (zero discharge with 100% salt harvesting). Although the strong harsh acidic conditions of FeCl_3_ solution, both woven and non-woven fabric showed a good resistant after washing and reuse again^56,57^.

The observed edge-preferential crystallization, which restricts salt accumulation to specific sites, suggests the feasibility of efficient in-situ salt harvesting. Prolonged evaporation resulted in the continuous crystallization of NaCl at the periphery of the evaporation disc, forming a suspended salt ring. The augmented mass from accumulating salt crystals induced a subtle deformation of the evaporation disc, suggesting that the adhesive forces between the crystals and the disc exceeded the gravitational pull on the crystals^53^. To assess the durability, photo-stability, and performance of the 0.1 M CuCl_2_-doped 10 μL pyrrole membrane, five consecutive tests were conducted using the previously mentioned four salt solutions. Evaporation and salt recovery rates were determined during these trials. Experiments were conducted using a 7.0% NaCl solution and a membrane surface area of 33.18 cm^2^. The measured evaporation rate was determined to be 0.84 kg m^− 2^ h^− 1^. Upon complete evaporation, the recovered salt was harvested, weighed, and quantified as 7.0 g, representing a 100% salt harvesting efficiency according to equation no 2.

2 $${\mathrm{salt}}\;{\mathrm{recovery}}\;{\mathrm{effeciency}} = \frac{Wt \cdot A}{{Wt \cdot B}} \times 100$$

where Wt. A is the weight of salt after complete evaporation of water and Wt. B is the weight of salt before dissolved in water.

A steady-state salt collection rate of approximately 58.60 g m^− 2^h^− 1^ was established based on gravimetric measurements of salt accumulation. Furthermore, a pilot experiments of simultaneous freshwater and salt harvesting were conducted using two A4-size non-woven photothermal membranes as explained in the supplementary file (Fig. S7 and S8).

Materials exhibiting poor wettability are susceptible to exacerbated membrane fouling during extended desalination processes ^58,59^. This phenomenon arises due to the propensity of such materials to accumulate significant salt deposits on their surfaces. In contrast, the developed photothermal membranes showed hydrophobicity with such fabric design of the top surface of the membrane led to simultaneous vapor and salt generation without pores blocking. The nonwoven fabric photothermal membranes showed a notable self-salt-repelling, specifically with NaCl, as shown in Fig. 14c, during evaporation under natural sunlight for 5 days. These phenomena suggest the continued solar desalination with simultaneous salts harvesting in different environments. The harvested salts of NaCl, FeCl_3_, and CuSO_4_.5H_2_O were investigated by XRD, as shown in Fig. S9. These observations highlight the influence of both solution concentration and membrane fouling on the evaporation rate during the simultaneous salts extraction and freshwater harvesting using the developed photothermal membranes.

## Conclusion

This study presented a facile, general, and environmentally-friendly chemical vapor deposition polymerization (CVDP) strategy for the fabrication of PPy-coated photothermal membranes. By evaluating the performance of membranes prepared using various oxidizing agents (i.e. FeCl_3_, CuCl_2_, (NH_4_)_2_S_2_O_8_, KMnO_4_, and Na_2_Cr_2_O_7_), where the most efficient oxidizing agent for PPy formation is identified with the aim of achieving optimal photothermal conversion efficiency. This approach offers a promising route for the development of robust, scalable, and sustainable sun-driven evaporation technologies for applications such as water desalination. The current investigation revealed that all the tested oxidizing agents (FeCl_3_, CuCl_2_, (NH_4_)_2_S_2_O_8_, KMnO_4_, and Na_2_Cr_2_O_7_) successfully initiated the polymerization and deposition of a dark PPy layer on various substrates, including woven fabric, non-woven polyester, PVDF, and cellulose nitrate. This demonstrates the versatility of the CVDP approach for PPy formation on diverse materials. However, the use of sodium dichromate (Na_2_Cr_2_O_7_) resulted in the deposition of a light brown layer on the substrate surface, potentially hindering the formation of a dense and dark PPy layer compared to the other oxidizing agents. Following PPy layer polymerization, the resulting PPy film exhibits a microstructured surface morphology. This morphology minimizes diffuse reflection, potentially enhancing light absorption by the photothermal membrane. The high average absorption of 94% within the 200–2400 nm range signifies the strong light-harvesting properties of these photothermal membranes. This broad spectral absorption stems from the intrinsic characteristics of the PPy layer, making the membranes efficient solar energy collectors for photothermal applications. The designed evaporation system enabled the PPy-coated membranes to achieve remarkable photothermal performance; that is, evaporation rates of 0.95 kg m^− 2^ h^− 1^ and 2.91 kg m^− 2^ h^− 1^ were recorded under irradiation equivalent to 1 and 3 sun, respectively, to assess its photo-stability. Evaporation rates of 0.95 kg m^− 2^ h^− 1^ and 2.91 kg m^− 2^ h^− 1^ were recorded under these conditions, respectively. These impressive results correspond to a solar conversion efficiency of 57%. These findings demonstrate the promising potential of PPy-coated membranes for efficient solar-driven water evaporation applications. The photothermal membranes displayed remarkable stability and maintained satisfactory evaporation performance across a range of challenging environments. This includes real seawater samples, wastewater, and even acidic or alkaline solutions. These findings suggest the potential broad applicability of these membranes for solar-driven desalination and wastewater treatment processes. Simultaneously, pure water and salt recovery was achieved, yielding a salt harvest rate of 58.6 g m^− 2^ h^− 1^ with 100% efficiency. The CVDP strategy presented here for PPy-coated membrane fabrication holds immense promise for widespread application in sustainable solar-driven clean water production technologies. This facile, versatile, and environmentally friendly approach offers a path towards efficient desalination and wastewater treatment processes, contributing significantly to addressing global water scarcity challenges.

## Supplementary Information

Supplementary Information.

## Data Availability

All data generated or analyzed during this study are included in this published article and its supplementary information file.

## References

[1] Oki, T. & Kanae, S. Global hydrological cycles and world water resources. *Science* **313**(5790), 1068–1072 (2006). 16931749 10.1126/science.1128845

[2] Schiermeier, Q. Water risk as world warms. *Nature* **505**(7481), 10 (2014). 24380936 10.1038/505010a

[3] Qadir, M., Jiménez, G. C., Farnum, R. L., Dodson, L. L. & Smakhtin, V. Fog water collection: challenges beyond technology. *Water* **10**(4), 372 (2018).

[4] Ibrahim, I., Seo, D. H., McDonagh, A. M., Shon, H. K. & Tijing, L. Semiconductor photothermal materials enabling efficient solar steam generation toward desalination and wastewater treatment. *Desalination* **500**, 114853 (2021).

[5] Liu, Y. et al. A bioinspired, reusable, paper-based system for high-performance large-scale evaporation. *Adv. Mater.* **27**(17), 2768–2774 (2015). 25809733 10.1002/adma.201500135

[6] Neumann, O. et al. Solar vapor generation enabled by nanoparticles. *ACS Nano* **7**(1), 42–49 (2013). 23157159 10.1021/nn304948h

[7] Ghasemi, H. et al. Solar steam generation by heat localization. *Nat. Commun.* **5**(1), 4449 (2014). 25043613 10.1038/ncomms5449

[8] Zhou, J. et al. Macroscopic and mechanically robust hollow carbon spheres with superior oil adsorption and light-to-heat evaporation properties. *Adv. Func. Mater.* **26**(29), 5368–5375 (2016).

[9] Ito, Y. et al. Multifunctional porous graphene for high-efficiency steam generation by heat localization. *Adv. Mater.* **27**(29), 4302–4307 (2015). 26079440 10.1002/adma.201501832

[10] Zhou, L. et al. 3D self-assembly of aluminium nanoparticles for plasmon-enhanced solar desalination. *Nat. Photonics* **10**(6), 393–398 (2016).

[11] Shi, Y. et al. A 3D photothermal structure toward improved energy efficiency in solar steam generation. *Joule* **2**(6), 1171–1186 (2018).

[12] Lu, Y., Zhang, H., Fan, D., Chen, Z. & Yang, X. Coupling solar-driven photothermal effect into photocatalysis for sustainable water treatment. *J. Hazard. Mater.* **423**, 127128 (2022). 34534804 10.1016/j.jhazmat.2021.127128

[13] Jiang, Q. et al. Bilayered biofoam for highly efficient solar steam generation. *Adv. Mater.* **28**(42), 9400–9407 (2016). 27432591 10.1002/adma.201601819

[14] Hua, Z. et al. Designing a novel photothermal material of hierarchical microstructured copper phosphate for solar evaporation enhancement. *J. Phys. Chem. C* **121**(1), 60–69 (2017).

[15] Wang, Y., Wang, C., Song, X., Megarajan, S. K. & Jiang, H. A facile nanocomposite strategy to fabricate a rGO–MWCNT photothermal layer for efficient water evaporation. *J. Mater. Chem. A* **6**(3), 963–971 (2018).

[16] Gao, X. et al. Synthesis of hierarchical graphdiyne-based architecture for efficient solar steam generation. *Chem. Mater.* **29**(14), 5777–5781 (2017).

[17] Yusuf Shi, Y. S., Li RenYuan, L. R., Shi Le, S. L., Elaf Ahmed, E. A., Jin Yong, J. Y., & Wang Peng, W. P. (2018). A robust CuCr2O4/SiO2 composite photothermal material with underwater black property and extremely high thermal stability for solar-driven water evaporation.‏

[18] Guo, H. X. et al. Evaporator fabricated with accessible photothermal material derived from waste fallen leaves for highly efficient desalination. *Appl. Surf. Sci.* **619**, 156728 (2023).

[19] Liu, H. et al. In situ polymerization of polypyrrole in oil body for efficient solar-driven freshwater collection. *Chem. Eng. J.* **468**, 143619 (2023).

[20] Liu, Z. et al. High-absorption recyclable photothermal membranes used in a bionic system for high-efficiency solar desalination via enhanced localized heating. *J. Mater. Chem. A* **5**(37), 20044–20052 (2017).

[21] Wang, Y. et al. Improved light-harvesting and thermal management for efficient solar-driven water evaporation using 3D photothermal cones. *J. Mater. Chem. A* **6**(21), 9874–9881 (2018).

[22] Zhang, L., Tang, B., Wu, J., Li, R., & Wang, P. (2015). Hydrophobic light-to-heat conversion membranes with self-healing ability for interfacial solar heating. 10.1002/adma.201502362 26184454

[23] Inzelt, G., & Inzelt, G. (2012). Chemical and electrochemical syntheses of conducting polymers. *Conduct. Polym. A New Era Electrochem.* pp 149–171‏

[24] Choy, K. L. Chemical vapour deposition of coatings. *Prog. Mater Sci.* **48**(2), 57–170 (2003).

[25] Bui, T. T., Kim, Y. S., Chun, H., La, D. D. & Bhosale, S. V. Template synthesis of micro/mesoporous Cl-doped polypyrrole using vapor phase polymerization. *Mater. Lett.* **192**, 80–83 (2017).

[26] Pedersen, H., Barry, S. T., & Sundqvist, J. (2021). Green CVD—Toward a sustainable philosophy for thin film deposition by chemical vapor deposition. *J. Vacuum Sci. Technol. A* **39**(5)

[27] Kumar Balu, S., Cheng, S., Latthe, S. S., Xing, R., & Liu, S. (2024). Solar-driven interfacial evaporation: Materials design and device assembly. *Energy Mater.* **4**(2)

[28] Yang, J., Cheng, H., Martens, W. N. & Frost, R. L. Transition of synthetic chromium oxide gel to crystalline chromium oxide: A hot-stage Raman spectroscopic study. *J. Raman Spectrosc.* **42**(5), 1069–1074 (2011).

[29] Han, J. et al. Full performance nanoporous graphene based Li-O2 batteries through solution phase oxygen reduction and redox-additive mediated Li2O2 oxidation. *Adv. Energy Mater.* **7**(7), 1601933 (2017).

[30] Kim, J., Sohn, D., Sung, Y. & Kim, E. R. Fabrication and characterization of conductive polypyrrole thin film prepared by in situ vapor-phase polymerization. *Synth. Met.* **132**(3), 309–313 (2003).

[31] Li, X. et al. Graphene oxide-based efficient and scalable solar desalination under one sun with a confined 2D water path. *Proc. Natl. Acad. Sci.* **113**(49), 13953–13958 (2016). 27872280 10.1073/pnas.1613031113 PMC5150409

[32] Wang, Z. et al. based membranes on silicone floaters for efficient and fast solar-driven interfacial evaporation under one sun. *J. Mater. Chem. A* **5**(31), 16359–16368 (2017).

[33] Ren, H. et al. Hierarchical graphene foam for efficient omnidirectional solar–thermal energy conversion. *Adv. Mater.* **29**(38), 1702590 (2017). 10.1002/adma.201702590 28833544

[34] Lou, J. et al. Bioinspired multifunctional paper-based rGO composites for solar-driven clean water generation. *ACS Appl. Mater. Interfaces.* **8**(23), 14628–14636 (2016). 27228106 10.1021/acsami.6b04606

[35] Huda, E. (2019). Preparation and characterization of cellulose acetate from cotton. In *IOP Conference Series: Earth and Environmental Science* (Vol. 364, No. 1, p. 012021). IOP Publishing.‏

[36] Bull, J. N., Maclagan, R. G., Fitchett, C. M. & Tennant, W. C. A new isomorph of ferrous chloride tetrahydrate: A 57Fe Mössbauer and X-ray crystallography study. *J. Phys. Chem. Solids* **71**(12), 1746–1753 (2010).

[37] Zuo, M. et al. Solventless preparation of ammonium persulfate microcapsules with a polypyrrole shell. *J. Mater. Sci.* **54**(7), 5898–5906 (2019).

[38] Jianu, O. A., Lescisin, M., Wang, Z., Rosen, M. A. & Naterer, G. F. X-ray diffraction of crystallization of copper (II) chloride for improved energy utilization in hydrogen production. *Int. J. Hydrogen Energy* **41**(19), 7848–7853 (2016).

[39] Zhu, H. et al. Concentration-dependent structure of mixed (NH4) 2SO4 and K2SO4 aqueous solutions using the X-ray diffraction, Raman spectroscopy and molecular dynamics simulations. *Vib. Spectrosc.* **116**, 103292 (2021).

[40] Datta, S. et al. Magnetic behavior and Raman spectroscopy of the composite system of CuCl2· 2H2O–C12H9NO. *J. Sci. Adv. Mater. Devices* **3**(1), 113–121 (2018).

[41] Dang, T. D., Banerjee, A. N., Joo, S. W. & Min, B. K. Effect of potassium ions on the formation of crystalline manganese oxide nanorods via acidic reduction of potassium permanganate. *Ind. Eng. Chem. Res.* **52**(39), 14154–14159 (2013).

[42] Subramanian, N., Viswanathan, B. & Varadarajan, T. K. A facile, morphology-controlled synthesis of potassium-containing manganese oxide nanostructures for electrochemical supercapacitor application. *RSC Adv.* **4**(64), 33911–33922 (2014).

[43] Planck, M. (1914). *The theory of heat radiation*. Blakiston.‏

[44] Wang, Y., Zhang, L. & Wang, P. Self-floating carbon nanotube membrane on macroporous silica substrate for highly efficient solar-driven interfacial water evaporation. *ACS Sustain. Chem. Eng.* **4**(3), 1223–1230 (2016).

[45] Qi, G., Wu, Z. & Wang, H. Highly conductive and semitransparent free-standing polypyrrole films prepared by chemical interfacial polymerization. *J. Mater. Chem. C* **1**(42), 7102–7110 (2013). 10.1039/c2cc33889k 22785579

[46] Navale, S. T., Mane, A. T., Ghanwat, A. A., Mulik, A. R. & Patil, V. B. Camphor sulfonic acid (CSA) doped polypyrrole (PPy) films: Measurement of microstructural and optoelectronic properties. *Measurement* **50**, 363–369 (2014).

[47] Yu, S. et al. The impact of surface chemistry on the performance of localized solar-driven evaporation system. *Sci. Rep.* **5**(1), 13600 (2015). 26337561 10.1038/srep13600 PMC4559801

[48] Xia, J., Chen, L. & Yanagida, S. Application of polypyrrole as a counter electrode for a dye-sensitized solar cell. *J. Mater. Chem.* **21**(12), 4644–4649 (2011).

[49] Zhu, J. et al. Polypyrrole metacomposites with different carbon nanostructures. *J. Mater. Chem.* **22**(11), 4996–5005 (2012).

[50] Shi, K. & Zhitomirsky, I. Polypyrrole nanofiber–carbon nanotube electrodes for supercapacitors with high mass loading obtained using an organic dye as a co-dispersant. *J. Mater. Chem. A* **1**(38), 11614–11622 (2013).

[51] Saafan, S. A., El-Nimr, M. K. & El-Ghazzawy, E. H. Study of dielectric properties of polypyrrole prepared using two different oxidizing agents. *J. Appl. Polym. Sci.* **99**(6), 3370–3379 (2006).

[52] Liu, F. et al. Synthesis of polypyrrole nanocomposites decorated with silver nanoparticles with electrocatalysis and antibacterial property. *Compos. B Eng.* **69**, 232–236 (2015).

[53] Zavvou, Z. (2023). *Initiated chemical vapour deposition of polymer thin films for power electronics* (Doctoral dissertation, Université Grenoble Alpes [2020-....]).‏

[54] Zhou, M. et al. Magnetic field induced the assembling of Photothermal evaporator for efficient solar-driven desalination. *EcoMat* **5**(9), e12390 (2023).

[55] Abdel-Ghafar, H. M., Song, X. & Jiang, H. Enhanced solar-driven evaporation process via f-MWCNTs/PVDF photothermal membrane for forward osmosis draw solution recovery. *Nanotechnology* **32**(37), 375703 (2021). 10.1088/1361-6528/ac084b 34087808

[56] Xu, Q. et al. Polypyrrole-coated cotton fabrics prepared by electrochemical polymerization as textile counter electrode for dye-sensitized solar cells. *Org. Electron.* **29**, 107–113 (2016).

[57] Wang, Y., Tao, S., An, Y., Wu, S. & Meng, C. Bio-inspired high performance electrochemical supercapacitors based on conducting polymer modified coral-like monolithic carbon. *J. Mater. Chem. A* **1**(31), 8876–8887 (2013).

[58] Irshad, M. S. et al. Advances of 2D-enabled photothermal materials in hybrid solar-driven interfacial evaporation systems toward water-fuel-energy crisis. *Adv. Func. Mater.* **33**(51), 2304936 (2023).

[59] Cha, H. et al. Dropwise condensation on solid hydrophilic surfaces. *Sci. Adv.* **6**(2), eaax0746 (2020). 31950076 10.1126/sciadv.aax0746 PMC6954056

[60] Pan, Q., Zhang, S., Li, R., He, Y. & Wang, Y. A low-cost and reusable photothermal membrane for solar-light induced anti-bacterial regulation. *J. Mater. Chem. B* **7**(18), 2948–2953 (2019).

[61] Guo, X., Gao, H., Wang, S., Yin, L. & Dai, Y. Scalable, flexible and reusable graphene oxide-functionalized electrospun nanofibrous membrane for solar photothermal desalination. *Desalination* **488**, 114535 (2020).

[62] Xia, Y. et al. Spatially isolating salt crystallisation from water evaporation for continuous solar steam generation and salt harvesting. *Energy Environ. Sci.* **12**(6), 1840–1847 (2019).

