# Supplementary material for: Development of robust dual functioning PPy-based photothermal membranes for simultaneous freshwater and salt harvesting
Source: Sci Rep. 2026 Feb 10;16:5945. doi: 10.1038/s41598-026-35812-y (PMC12894873; doi:10.1038/s41598-026-35812-y)
Supplement: Supplementary file 1 — Supplementary Information. [file 41598_2026_35812_MOESM1_ESM.docx]

**Development of Robust Dual Functioning PPy-based Photothermal Membranes for Simultaneous Freshwater and Salts Harvesting**

Mahmoud Taha Mahmoud^1, 2^, Hamdy Maamoun Abdel-Ghafar^1^^[[1]](#footnote-1)^*, Ahmed Abdou El-Sherif^2^, Mohamed Saada El-Deab^2*^

^1^Central Metallurgical Research and Development Institute (CMRDI), PO Box 87 Helwan, Cairo, Egypt

^2^Department of Chemistry, Faculty of Science, Cairo University, 12613 Cairo, Egypt

1. **Optimizing the oxidizing agents on different fabrics (substrates)**


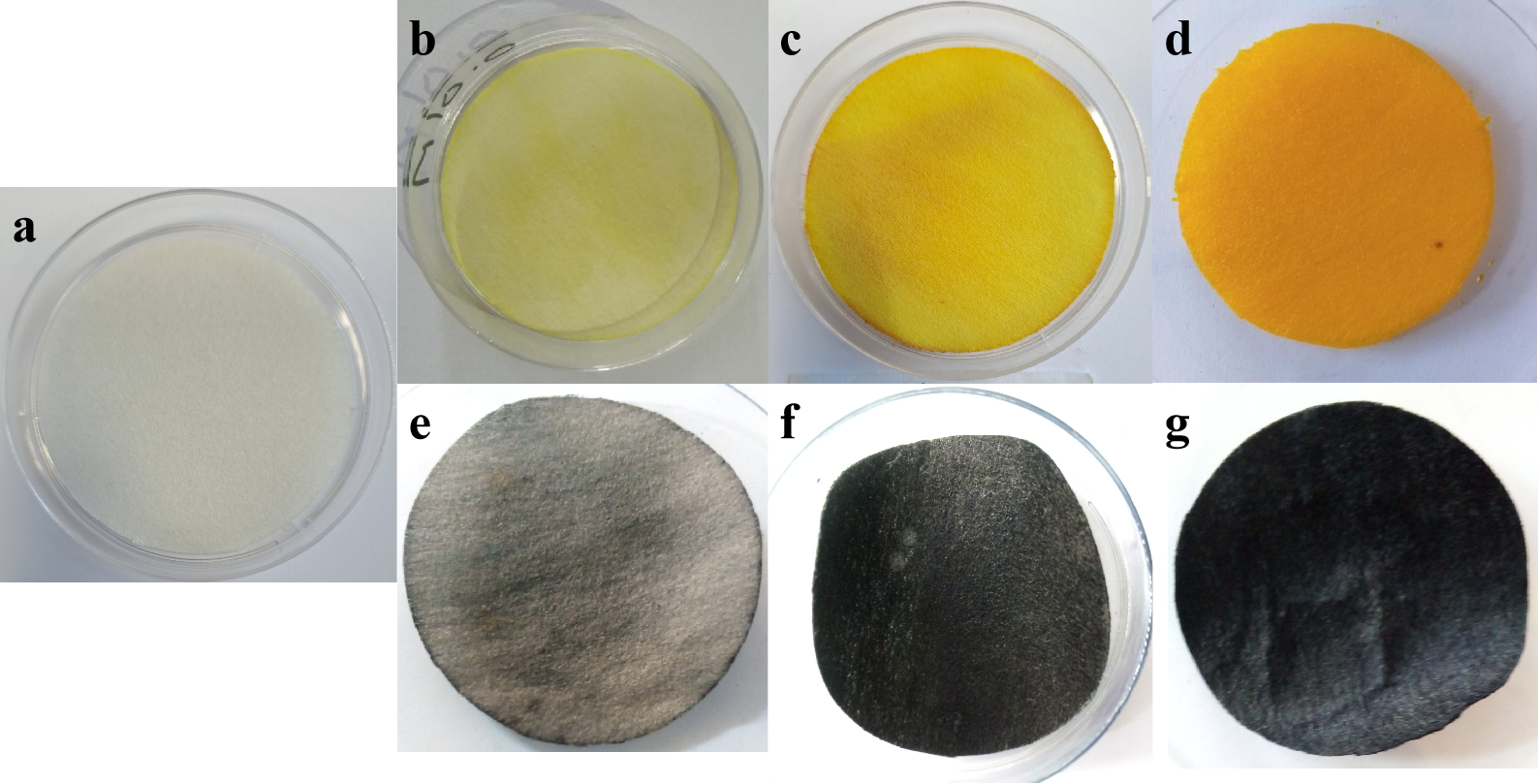


**Fig. S1.** a) non-woven blank, b) 0.01M FeCl_3_, c) 0.1 M, d) 0.5 M, e, f & g 15 μL pyrrole (py) of b-d at 80 °C


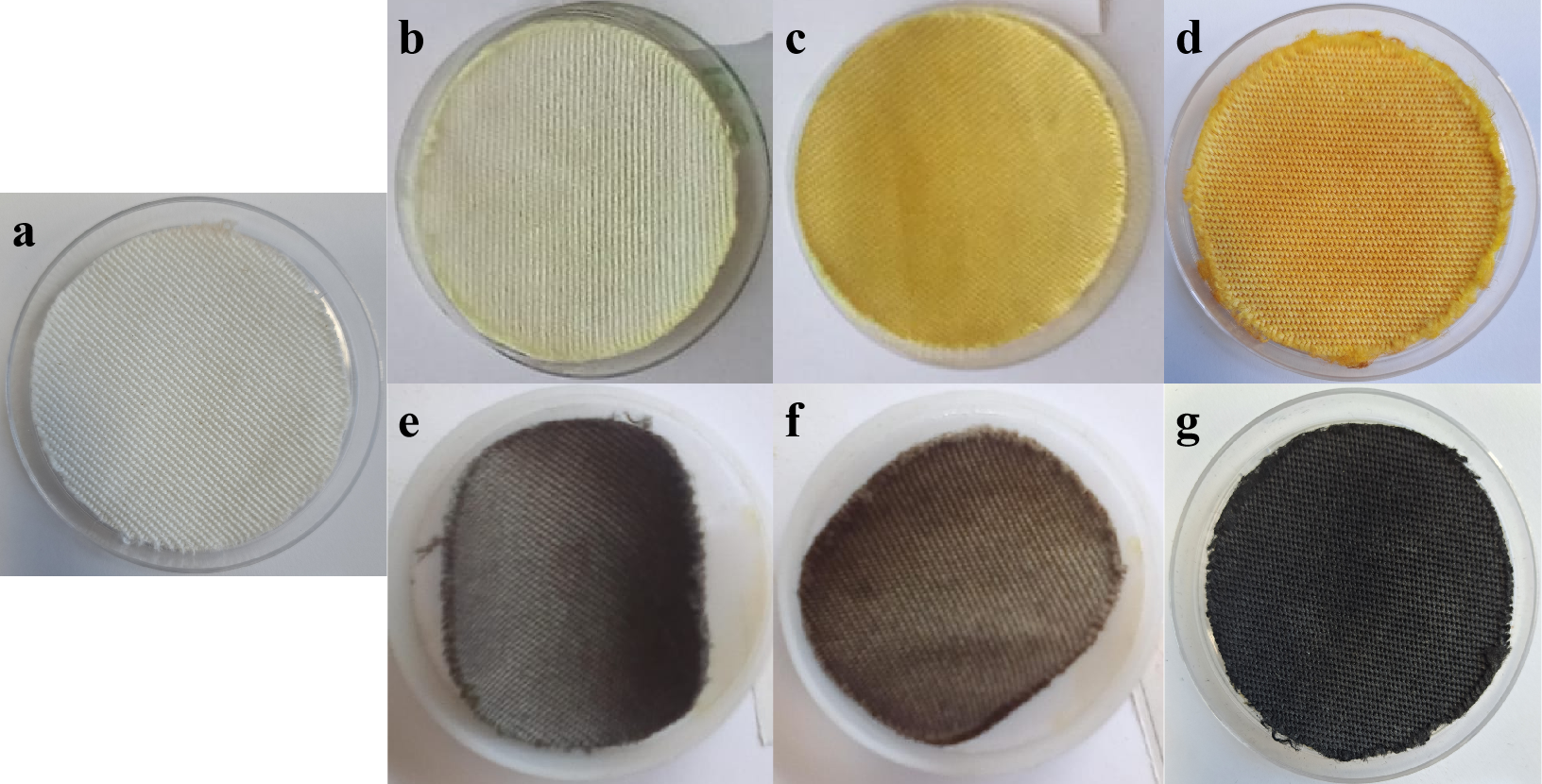


**Fig. S2.** a) woven blank, b) 0.01M FeCl_3_, c) 0.1 M, d) 0.5 M, e & f 20 μL, while g 15 μL pyrrole at 80 °C


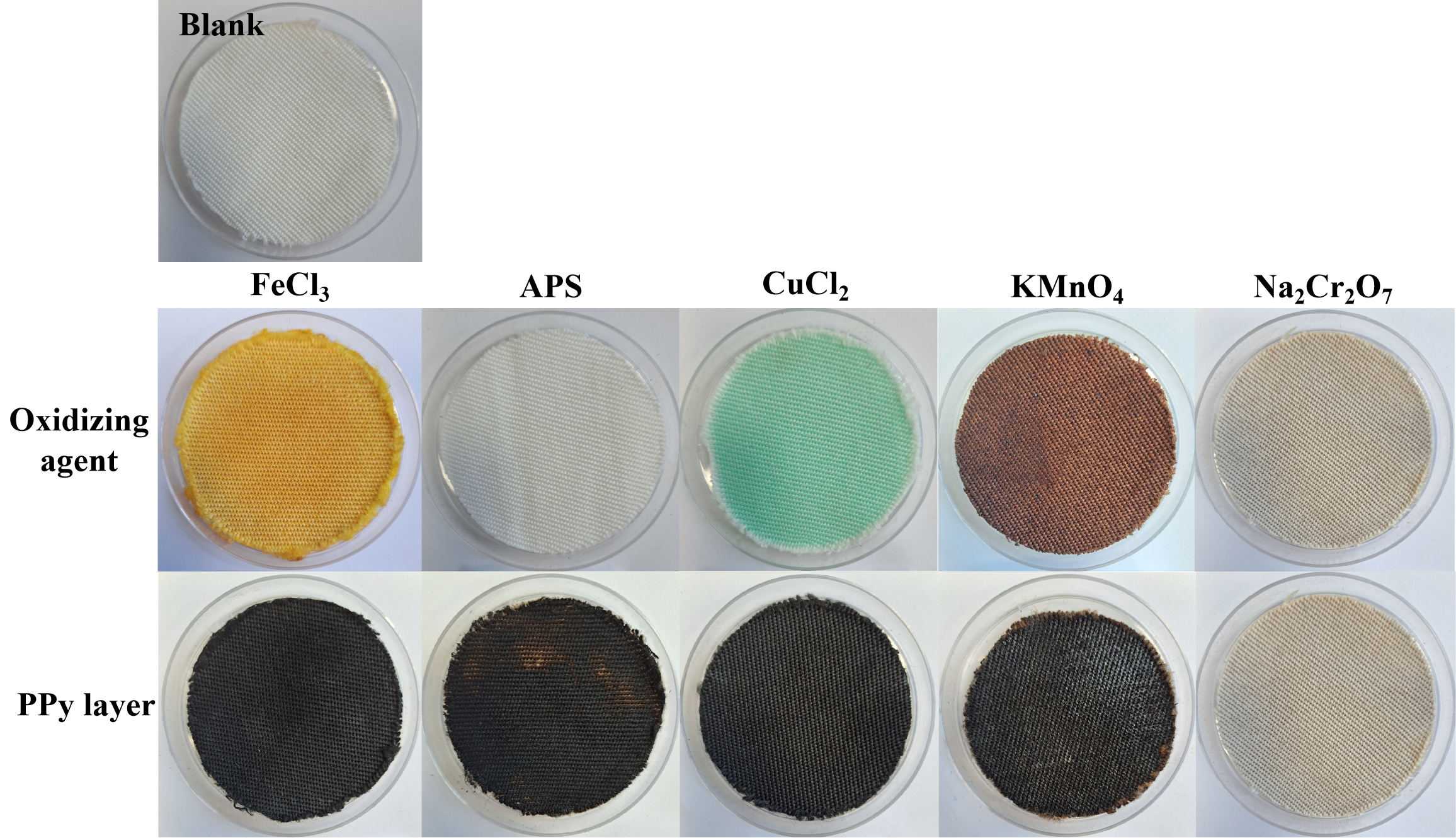


**Fig. S3.** Blank “woven” - (0.5 M) FeCl_3_ (15 uL py) - APS (20 uL py) - CuCl_2_ (15 uL py) - KMnO_4_ (20 uL py) - Na_2_Cr_2_O_7_ (20 uL py)


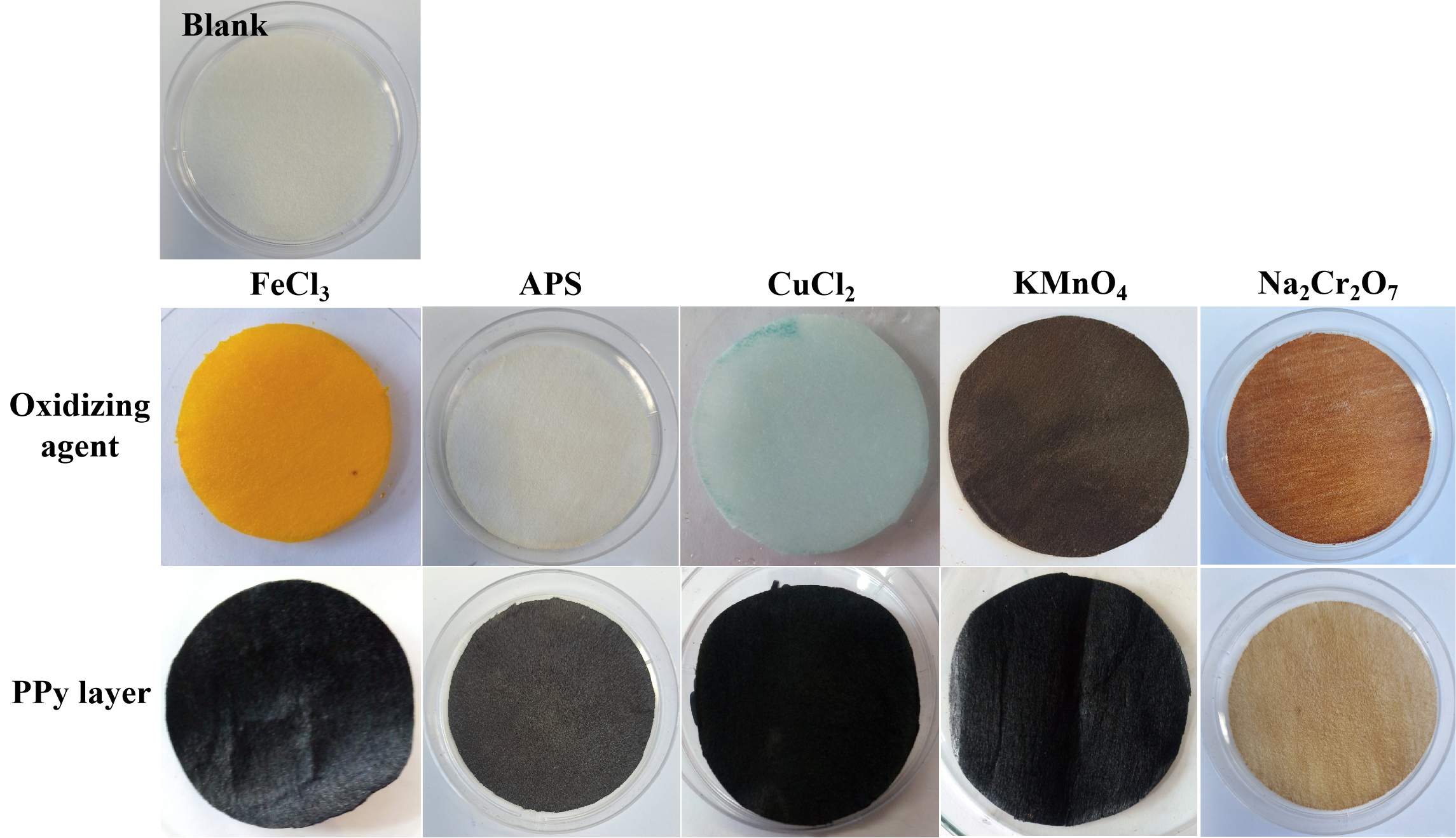


**Fig. S4.** Blank “non-woven” - (0.5 M) FeCl_3_ (15 uL py) - (0.1 M) APS (15 uL py) - (0.5 M) CuCl_2_ (15 uL py) - (0.1 M) KMNO_4_ (15 uL py of 0.5M) - (0.5 M) Na_2_Cr_2_O_7_ (20 uL py)

1. **Optimizing chemical vapor deposition polymerization (CVDP) of polypyrrole (PPy):**

Polypyrrole (PPy) is synthesized primarily through oxidative polymerization, employing either chemical or electrochemical methods. Chemical oxidation typically involves the use of oxidants such as ferric chloride, ammonium persulfate, potassium persulfate, or copper (II) chloride to induce pyrrole polymerization. While chemical oxidation is commonly employed, it is important to note that pyrrole deposition can also be achieved via chemical vapor deposition polymerization (CVDP) [1, 2]. CVDP is a versatile and straightforward technique for producing polymeric coatings under mild conditions. Notably, pyrrole monomers can effectively polymerize within the porous structures of substrates and metal-organic frameworks (MOFs) through vapor-phase reactions [3]. The properties of PPy coatings are influenced by factors such as the type and concentration of oxidant, polymerization temperature, duration, and substrate characteristics. This study investigates the interplay between different substrates, oxidants, and feed solutions in the development of PPy coatings via CVDP for potential applications in freshwater, salt extraction and harvesting.

1. **Simultaneous salts extraction and freshwater harvesting**

Photothermal systems have emerged as a potential solution to global water scarcity, utilizing solar energy to effectuate water evaporation. The effective condensation of generated vapor into potable water is a critical factor in these systems' performance [4, 5]. Condensation is a physical process whereby water vapor undergoes a phase transformation to liquid water. In photothermal systems, this transition is facilitated by the dissipation of energy from vapor molecules, enabling droplet formation [6]. One of the objectives of this research is to purify water from salt solutions via photothermal membrane evaporation, with simultaneous salts recovery. The integration of a condensation module facilitates the conversion of steam into collectible, purified water.

When designing a salt extraction process using evaporation, the initial concentration of the solution is a crucial factor. Saltier solutions inherently have slower evaporation rates. This can impact process efficiency and may require adjustments like increased surface area or longer evaporation times. Salt accumulation on the membrane needs to be addressed to maintain efficient evaporation. Strategies like using smoother membrane surfaces, choosing materials less prone to salt adhesion, or incorporating periodic cleaning cycles can be explored to minimize fouling and maintain consistent evaporation rates.

By optimizing salt concentration and evaporation disc dimensions, salt crystallization was confined to the container's periphery, maintaining an unobstructed evaporative surface. These results suggest that this innovative solar steam generator possesses the potential for broader application in steam generation and salt recovery from diverse saline sources.

1. **Porosity of the photothermal membranes**

The dry-wet weight method was employed to quantify the porosity of the fabricated membranes based on their water content. The membranes were first hydrated in distilled water for one hour. Surface moisture was removed by blotting with filter paper, and the hydrated weight (Ww) was recorded. Subsequently, the samples were dried in an oven at 80 °C for 24 hours to achieve a constant dry weight (Wdr) [7]. The porosity P (%) was evaluated using equation (1).

$P \%=\frac{(Ww-Wdr)}{\mathrm{Ww}}\times100$ Eq.1

where; Ww is the weight of wet membrane and Wdr is the weight of dry membrane.

A comparative analysis of the photothermal membranes before and after polypyrrole (PPy) deposition, presented in Fig. S5, indicates a significant reduction in porosity post-polymerization, where the porosity was 78.6, 76.7, 62.3 and 60.7% for non-woven and woven fabric before and after deposition of PPy. The observed reduction is likely due to the hydrophobicity of the PPy layer, which modifies the membrane's microstructure, thereby reducing pore accessibility and overall porosity.


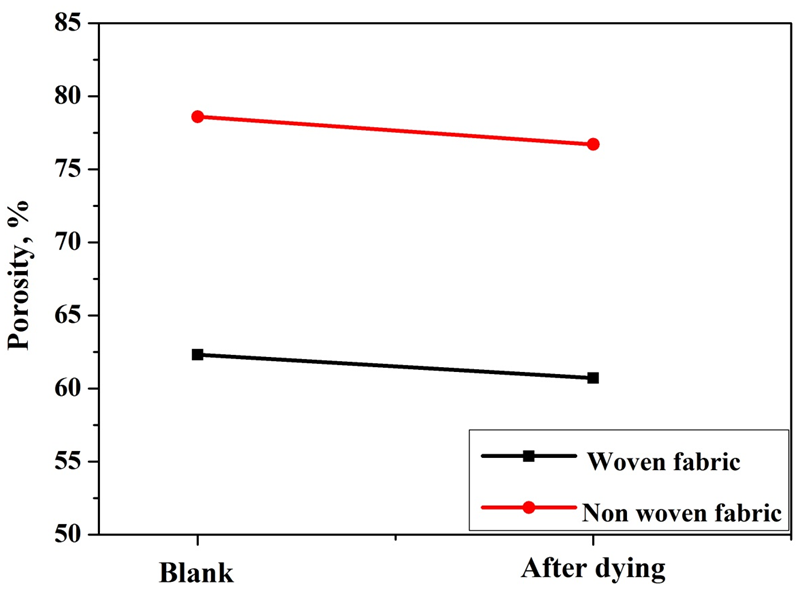


**Fig. S5** Porosity of Substrate Non-woven and woven before and after deposition of PPy.

1. **Performance evaluation of the substrates by black ink dye**


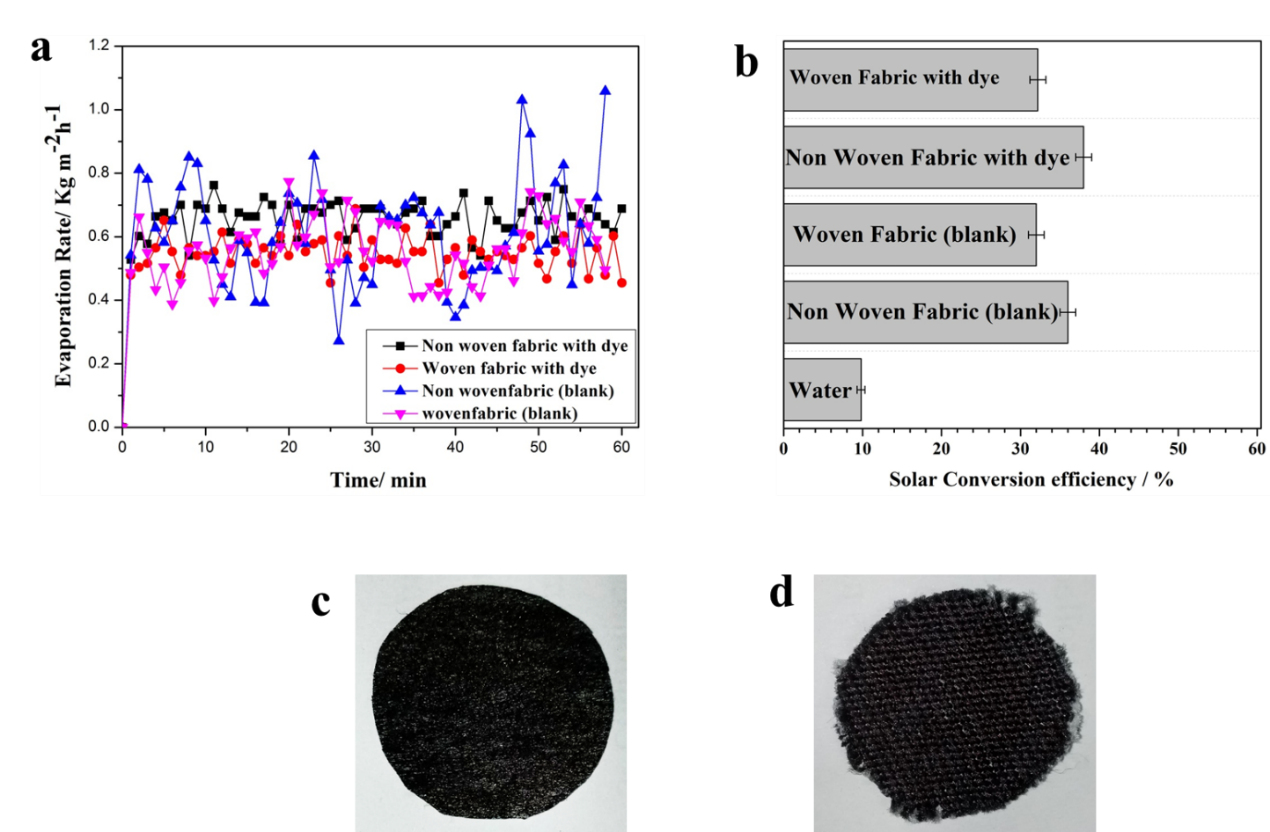


**Fig.S6** (a) Evaporation Rate of Substrate Non-woven and woven before and after coating with black ink dye. (b) Solar Thermal conversion efficiency of Substrate Non-woven and woven before and after coating with black ink dye, (c,d) Non-Woven and Woven image after coating with black dye ink, respectively.

1. **Pilot experiment set-up and evaluation**

To demonstrate the previously achieved work by the solar simulator, a pilot experiment was conducted at the CMRDI Institute in March 2024. Specifically, a test was conducted during the period from 4^th^ to 10^th^ March 2024 using 4 L of bitter water obtained from Borg Al Arab Salines Company, Alexandria, Egypt. At the end of the experiment (after 5 days), the collected pure water was about 1.66 L, and crystallized (precipitated) salts were about 459.4 g, as shown in **Fig. S7 and S8**. By considering the exposed photothermal surface area of the pilot experiment (18 cm × 45 cm = 0.081 m^2^). The pilot system was run for 5 days, where the average sunny period per day is approximately 8 h; therefore, the achieved evaporation rate by the pilot system under natural sunlight is 0.49 kg/m^2^h.

This reduction in evaporation rate under natural sunlight, compared to the results obtained using the solar simulator, may be attributed to the fact that natural sunlight is not consistently equivalent to 1 sun throughout the day and changes from morning to noon and before sunrise, in addition to the weather conditions around the experiments such as wind and dust that may form a shadow on the experimental and reduce the intensity of the incident sunlight, as shown in **Fig. S7 and S8**.


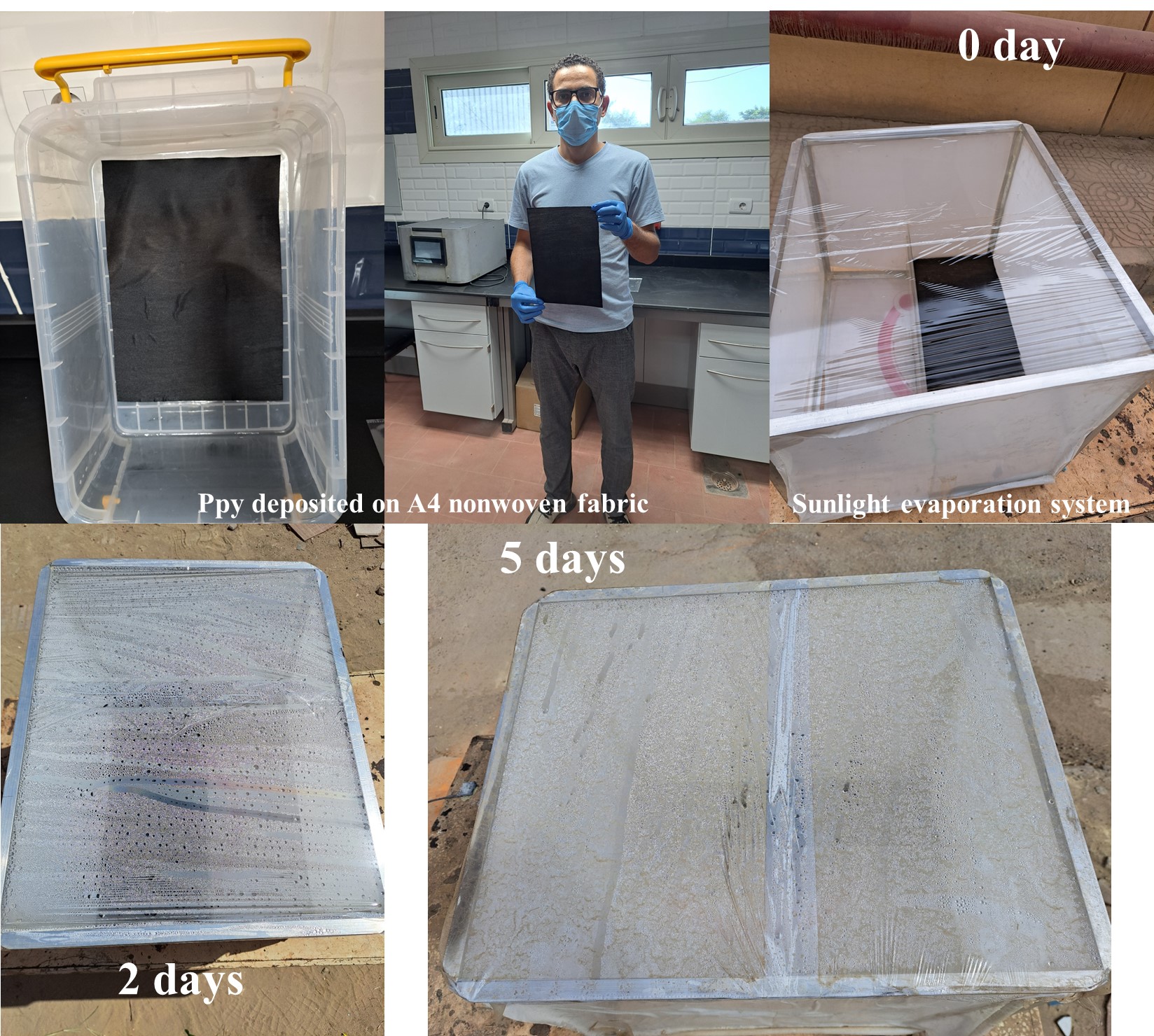


**Fig. S7.** a photograph of the prepared A4 size non-woven ppy photothermal membrane, and the custom-made evaporation system under natural sunlight for a few days using two A4-size photothermal membranes.


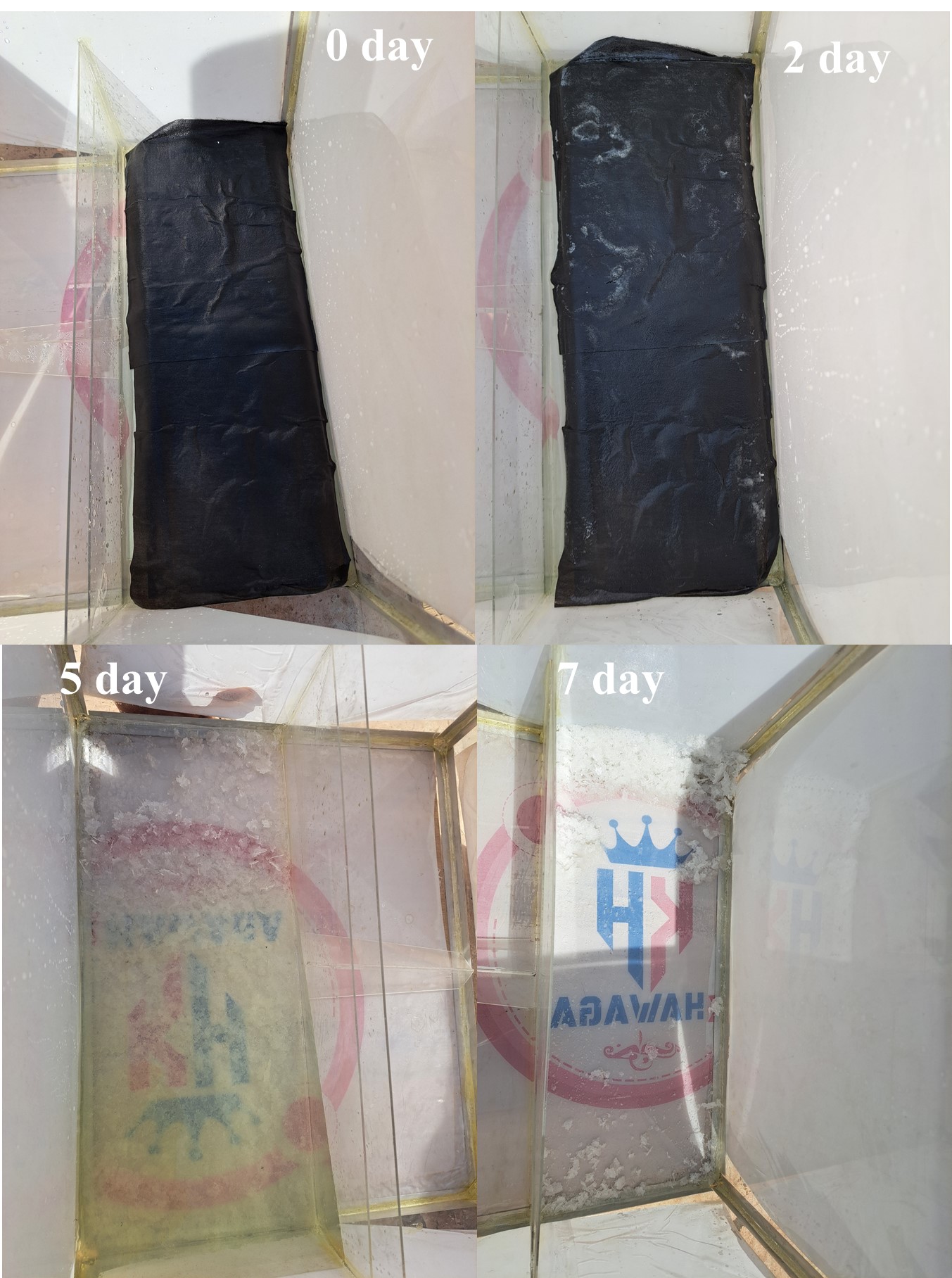


**Fig. S8.** The illuminated A4-size photothermal membranes in the pilot system at the beginning (0 day) and after two days of the experiment, and the precipitated salts after the end of the experiment at 5 and 7 days with complete evaporation.

1. **Simultaneous salts extraction and freshwater harvesting**

Following the complete evaporation of the water, salt crystals that formed on the bottle's edge were harvested and characterized using X-ray diffraction (XRD). The X-ray diffraction pattern exhibits characteristic peaks at 2θ values of 27.38°, 31.73°, 45.44°, 53.86°, and 56.47°, which are indexed to the salt crystal structure of NaCl [8]. The diffraction peaks observed at 22.22°, 29.18°, 32.06°, 36.81°, and 42.18° (2θ) correspond to the characteristic lattice planes of crystalline copper (II) sulfate pentahydrate (CuSO₄·5H₂O) [9]. The X-ray diffraction pattern exhibits distinct peaks at 2θ = 11.27°, 26.32°, 35.58°, 43.28°, 45.51°, 50.48°, and 64.16°, which are indexed to the crystalline structure of iron oxychloride (FeOCl). The presence of hydrated ferroxy chloride (FeOCl) is observed, resulting from the adsorption of atmospheric water vapor [10].


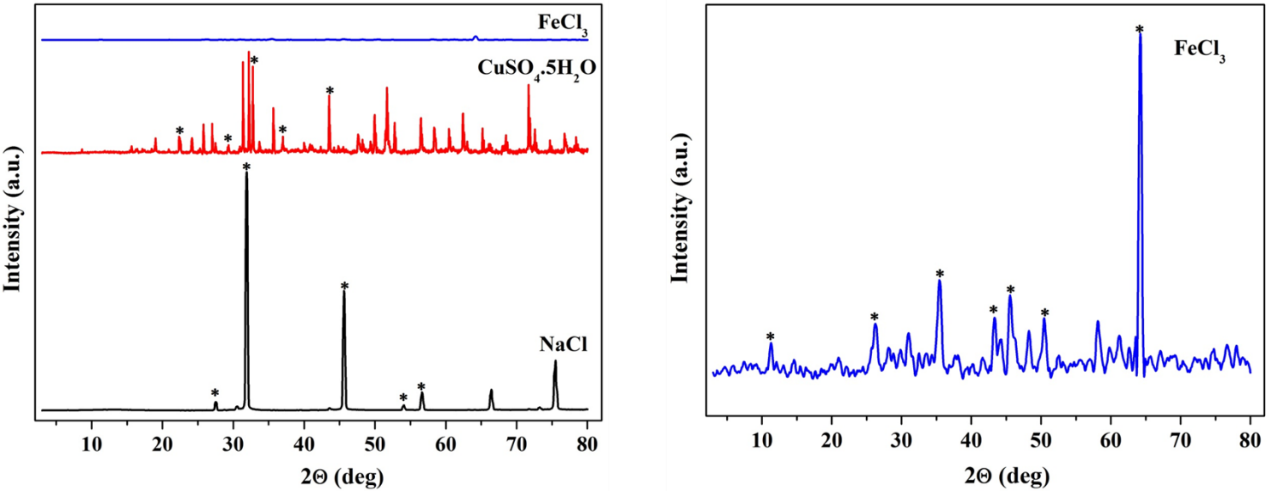


**Fig. S9.** XRD spectra of crystalized salt after complete evaporation of water.

**Table S1.** Comparison of the previous work with this study

| **Photothermal membrane types** | **Composition/ preparation** | **Evaporation rate**  **(Kg.m^-2^.h^-1^)** | **Other functions** | **Conversion efficiency, %** | **Ref.** |
| --- | --- | --- | --- | --- | --- |
| Filter paper | Deposited filterable [MXene](https://www.sciencedirect.com/topics/materials-science/mxene) | 1.34 | N/A | 81.5 | [11] |
| Nylon membrane | N_2_-doped CNTs encapsulating Co nanoparticles | 1.55 | dyeing sewage and heavy metal wastewater treatment | 89.7 | [12] |
| PANI nanofiber array | MOF/PANI nanofiber array | 1.0 | VOCs removal | -- | [13] |
| Commercial woven & non-woven fabric | PPy deposition | 0.95 | Salts harvesting | 57 | This work |

**References:**

[1] Irshad, M. S., Arshad, N., Asghar, M. S., Hao, Y., Alomar, M., Zhang, S., ... & Zhang, H. (2023). Advances of 2D‐enabled photothermal materials in hybrid solar‐driven interfacial evaporation systems toward water‐fuel‐energy crisis. *Advanced Functional Materials*, *33*(51), 2304936.‏

[2] Cha, H., Vahabi, H., Wu, A., Chavan, S., Kim, M. K., Sett, S., ... & Miljkovic, N. (2020). Dropwise condensation on solid hydrophilic surfaces. *Science advances*, *6*(2), eaax0746.‏

[3] Pan, Q., Zhang, S., Li, R., He, Y., & Wang, Y. (2019). A low-cost and reusable photothermal membrane for solar-light induced anti-bacterial regulation. *Journal of Materials Chemistry B*, *7*(18), 2948-2953.‏

[4] Guo, X., Gao, H., Wang, S., Yin, L., & Dai, Y. (2020). Scalable, flexible and reusable graphene oxide-functionalized electrospun nanofibrous membrane for solar photothermal desalination. *Desalination*, *488*, 114535.

[5] Xia, Y., Hou, Q., Jubaer, H., Li, Y., Kang, Y., Yuan, S., ... & Zhang, X. (2019). Spatially isolating salt crystallisation from water evaporation for continuous solar steam generation and salt harvesting. *Energy & Environmental Science*, *12*(6), 1840-1847.

[6] Saafan, S. A., El‐Nimr, M. K., & El‐Ghazzawy, E. H. (2006). Study of dielectric properties of polypyrrole prepared using two different oxidizing agents. *Journal of applied polymer science*, *99*(6), 3370-3379.

[7] Li, Z. Reliability Comparison of Wet-Dry Weight Methods for Membrane Porosity Measurement: Advantages and Limitations of Density Method Versus Area Method.

[8] Ahmad, K., Kakakhel, M. B., Hayat, S., Wazir-ud-Din, M., Mahmood, M. M., Ur Rehman, S., ... & Mirza, S. M. (2021). Thermoluminescence study of pellets prepared using NaCl from Khewra Salt Mines in Pakistan. Radiation and Environmental Biophysics, 60(2), 365-375.

[9] Ahemad, H. I., Patil, G. E., Shinde, S. D., Patil, D. Y., Jain, G. H., Kajale, D. D., ... & Patil, S. B. (2024). Synthesis and Characterization of Copper Sulfate Single Crystals. Physics of the Solid State, 66(12), 642-652.

[10] Kanungo, S. B., & Mishra, S. K. (1996). Thermal dehydration and decomposition of FeCl3· xH2O. Journal of thermal analysis, 46(5), 1487-1500.

[11] Su, J., Xie, Y., Zhang, P., Yang, R., Wang, B., Zhao, H., Xu, Y., Lin, X., Shi, J., Wang, C., Janus MXene-based photothermal membrane for efficient and durable water evaporation, *Desalination* 566, 2023, 116905.

[12] [Jiang, J.,](https://www.sciencedirect.com/author/7404830364/jing-jiang)Jiang, H., Xu, Y., Chen, M., [Ai,](https://www.sciencedirect.com/author/23994986700/lunhong-ai)L., Janus Co@C/NCNT photothermal membrane with multiple optical absorption for highly efficient solar water evaporation and wastewater purification, [*Colloids and Surfaces A: Physicochemical and Engineering Aspects*](https://www.sciencedirect.com/journal/colloids-and-surfaces-a-physicochemical-and-engineering-aspects) *647,* 2022, 128960.

[13] Peng, Y., Wei, X., Wang, Y., Li, W., Zhang, S**.,** Jin, J**.,** Metal–Organic Framework Composite Photothermal Membrane for Removal of High-Concentration Volatile Organic Compounds from Water via Molecular Sieving, *ACS Nano 16(5),* 2022, 8329–8337.

1. * **The corresponding authors:** Hamdy Maamoun Abdel-Ghafar, E-mail addresses: [hamdy.maamoun@gmail.com](mailto:hamdy.maamoun@gmail.com), [msaada68@yahoo.com](mailto:msaada68@yahoo.com) (M. S. El-Deab) [↑](#footnote-ref-1)
